# Multi-region sequencing unveils novel actionable targets and spatial heterogeneity in esophageal squamous cell carcinoma

**DOI:** 10.1038/s41467-019-09255-1

**Published:** 2019-04-11

**Authors:** Ting Yan, Heyang Cui, Yong Zhou, Bin Yang, Pengzhou Kong, Yingchun Zhang, Yiqian Liu, Bin Wang, Yikun Cheng, Jiayi Li, Shixing Guo, Enwei Xu, Huijuan Liu, Caixia Cheng, Ling Zhang, Ling Chen, Xiaofei Zhuang, Yu Qian, Jian Yang, Yanchun Ma, Hongyi Li, Fang Wang, Jing Liu, Xuefeng Liu, Dan Su, Yan Wang, Ruifang Sun, Shiping Guo, Yaoping Li, Xiaolong Cheng, Zhihua Liu, Qimin Zhan, Yongping Cui

**Affiliations:** 1 Shenzhen Peking University-The Hong Kong University of Science and Technology (PKU-HKUST) Medical Center, Peking University Shenzhen Hospital, 518035 Shenzhen, PR China; 2 Department of Pathology & Shanxi Key Laboratory of Carcinogenesis and Translational Research on Esophageal Cancer, Shanxi Medical University, 030001 Taiyuan, PR China; 3 Department of Tumor Surgery, Shanxi Cancer Hospital, 030013 Taiyuan, PR China; 4 College of Information and Computer, Taiyuan University of Technology, 030001 Taiyuan, PR China; 5 College of Letter & Science, University of California Berkeley, Berkeley, CA 94704 USA; 6 Anglo-Chinese School (Independent), Singapore, 139650 Singapore; 7 Department of Pathology, Shanxi Cancer Hospital, 030013 Taiyuan, PR China; 8 Department of Pathology, the First Hospital, Shanxi Medical University, 030001 Taiyuan, PR China; 9 Department of Pathology, Tianjin Central Hospital of Gynecology Obstetrics, Nankai University Gynecology Obstetrics Hospital, 300052 Tianjin, PR China; 10 Department of General Surgery, the First Hospital, Shanxi Medical University, 030001 Taiyuan, PR China; 11 Department of Pathology, Zhejiang Cancer Hospital, 310022 Hangzhou, PR China; 12 Key laboratory of Carcinogenesis and Translational Research (Ministry of Education/Beijing), Laboratory of Molecular Oncology, Peking University Cancer Hospital & Institute, 100191 Beijing, PR China; 13 Tumor Biobank, Shanxi Cancer Hospital, 030013 Taiyuan, PR China; 14 Department of Colorectal & Anal Surgery, Affiliated Provincial Hospital of Shanxi Medical University, 030001 Taiyuan, PR China; 15 State Key Laboratory of Molecular Oncology, National Cancer Center/Cancer Hospital, Chinese Academy of Medical Sciences and Peking Union Medical College, 100021 Beijing, PR China

## Abstract

Esophageal squamous cell carcinoma (ESCC) ranks fourth among cancer-related deaths in China due to the lack of actionable molecules. We performed whole-exome and T-cell receptor (TCR) repertoire sequencing on multi-regional tumors, normal tissues and blood samples from 39 ESCC patients. The data revealed 12.8% of *ERBB4* mutations at patient level and functional study supported its oncogenic role. 18% of patients with early *BRCA1/2* variants were associated with high-level contribution of signature 3, which was validated in an independent large cohort (*n* = 508). Furthermore, knockdown of *BRCA1/2* dramatically increased sensitivity to cisplatin in ESCC cells. 5% of patients harbored focal high-level amplification of *CD274* that led to massive expression of PD-L1, and might be more sensitive to immune checkpoint blockade. Finally, we found a tight correlation between genomic and TCR repertoire intra-tumor heterogeneity (ITH). Collectively, we reveal high-level ITH in ESCC, identify several potential actionable targets and may provide novel insight into ESCC treatment.

## Abstract (web summary)

Esophageal squamous cell carcinoma (ESCC) is highly prevalent in China. Here, the authors carry out multi-region sampling of Chinese ESCC samples, and find recurrent *ERBB4* mutations, *BRCA1/2* variants, and amplification of *CD274*; together with high levels of genomic and T-cell receptor heterogeneity.

## Introduction

Esophageal squamous cell carcinoma (ESCC) constitutes the predominant histology of esophageal cancer in China and has limited therapeutic options. Several studies revealed that tumors with high burden of *BRCA1/2* signature were more sensitive to platinum therapy in pancreatic and breast cancer^1,2^, which raises a possibility that a similar mutational pattern in ESCC may facilitate treatment for inoperable patients. Moreover, selecting drugs based on genomics data has led to promising results in early studies on personalized and targeted therapy. Some of targeted drugs have been utilized in ESCC, such as Gefitinib^3^ (an EGFR inhibitor), Dovitinib^4^ (an FGFR1 inhibitor), and PD-L1 blockers^5^. However, only a small subset of patients has long-term benefit from these target therapies, which raises the question whether the substantial intra-tumor heterogeneity (ITH) of cancer cells or tumor immune microenvironment such as tumor-infiltrating lymphocytes and derived T cell receptor (TCR) repertoire are responsible for the differences. Therefore, it is necessary to identify novel targets and assess the spatial heterogeneity of targets before decision making.

At present, large-scale whole-exome sequencing (WES) on ESCC was performed by several institutes^6–13^, which unveiled high frequency of mutations and mutational process. Nevertheless, most of them described ESCC mutational landscape using single-biopsy that might underestimate ITH and genomic evolution. ESCC evolution is a multi-step process that begins from simple hyperplasia or dysplasia, precursor lesion, to primary and metastatic tumor^14^. Recent literatures portrayed the genomic dynamics during these processes in ESCC^15,16^. Using multi-region sequencing, they confirmed early mutational events of mutations in *TP53,* *CDKN2A* and copy number alterations (CNAs) in 11q (*CCND1*), 3q (*SOX2*), 9p (*CDKN2A*), and identified sharply genomic changes between simple hyperplasia and dysplasia, of which simple hyperplasia harbored no or very low number of mutations. In contrast, most of CNAs were shared between dysplasia and ESCC. Although subclonal diversification within primary tumor was described by Hao et al.^17^ in 13 ESCC patients, the conclusion was relatively limited due to small sample cohort. Moreover, evolution from primary tumor to metastasis was studied to a lesser extent in these studies.

To overcome these challenges and comprehensively characterize the mutational landscape and ITH of ESCC at patient level, we applied multi-region exome sequencing (Mseq) on samples covering quinquefid primary tumors to metastatic lymph nodes derived from 39 patients (referred as 39-Mseq cohort), of which 10 patients were subjected to TCR repertoire sequencing. We found several potential actionable targets for a subset of ESCC patients, and revealed high-level heterogeneity of druggable targets or biomarkers among samples from different regions of the same ESCC patient, highlighting the necessity of multi-region sequencing for precise therapy.

## Results

### Mutational landscape of ESCC at patient level

**Fig. 1:**
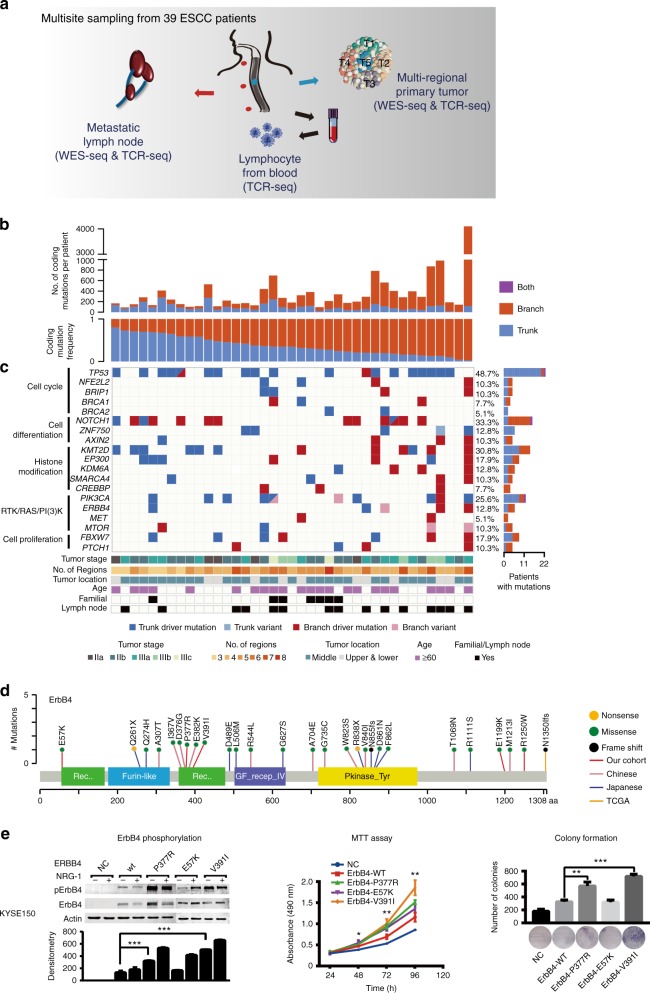
Heterogeneity of driver mutations across 39 ESCC individuals. **a** Sequencing and analytical pipeline in this study. Multisite samples including metastatic lymph nodes, lymphocytes, and primary tumors. Primary tumors were divided into five regions for each patient. **b** The top panel shows the number of coding mutations detected in each individual; the bottom panel shows the percentages of trunk or branch coding mutations. **c** Heat map shows somatic driver mutations with different clonal status. The right panel shows the proportion of the variants found as trunk/branch in ESCC; the bottom panel shows key clinicopathological characteristics. **d** Schematics of ErbB4 protein alterations resulted from mutations identified from the 39-Mseq ESCCs and additional TCGA, early Chinese and Japanese cohorts. **e** The effect of *ERBB4* wild type and mutants on ErbB4 phosphorylation and cell proliferation as monitored by NRG-1 stimulation (left), MTT (middle), and colony formation assay (right), respectively. Experiments were performed with KYSE150 cells in triplicate and all data are mean ± SD. ^*^P *<* 0.05, ^**^P *<* 0.01, ^***^P *<* 0.001. Source data are provided as a Source Data file

We sequenced 185 tumor regions (164 regions from primary tumors and 21 metastatic lymph nodes) and 39 matched normal esophageal tissues (5 regions per patient) from 39 patients, with an average sequencing depth of 290× for tumors and 187× for normal samples (Fig. 1a, Supplementary Figs. 1, 2a, b, Supplementary Data 1). We detected a total of 11,703 unique non-silent nucleotide substitutions and 961 indels, with a median of 189 non-silent substitutions (range: 67–3028) and 14 indels (range: 4–67) for each case (Supplementary Data 2). Mutations identified from three samples were further validated with an average of 98% accuracy (Methods, Supplementary Data 2b). The mutation number varied substantially among patients, especially for ESCC012, which exhibited extremely high mutation rate (Supplementary Fig. 2c). Moreover, mutation burden was higher in ESCC patients harboring larger tumor volume than those with smaller tumor (Wilcoxon rank sum test, *P* = 0.068, Supplementary Fig. 2d). At patient level, mutations per patient identified by multi-region sequencing were significantly greater than those with single-regional analysis or the mutation data from The Cancer Genome Atlas (TCGA)^18^ (Wilcoxon rank sum test, *P* = 0.021 and *P* = 0.037, respectively, Supplementary Fig. 2e).

To assess ITH in ESCC, we characterized tumor evolution as a phylogenetic tree structure (Methods) with the trunk representing ubiquitous mutations that present in all tumor regions, the shared branches representing heterogeneous mutations that present in some but not all tumor regions, and the private branches representing mutations that present in only one tumor region^19^. Notably, all 39 ESCCs exhibited a presence of spatial ITH, with an average of 63.2% somatic variants being heterogeneous (Fig. 1b). These results suggested that the majority of mutations occurred after carcinoma transformation. We found no correlations among the heterogeneity level and patient’s age, number of biopsies and genome doubling (Supplementary Fig. 3a–c). However, tumor stage negatively correlated with the proportion of ubiquitous mutations (Supplementary Fig. 3d), and significantly more heterogeneous mutations were observed in patients with metastatic lymph nodes (Wilcoxon rank sum test, *P* = 0.039, Supplementary Fig. 3e).

We identified 581 driver events (median: 10, range: 3–119) based on previously reported criteria^20^, and 181 of them were trunk mutations (Supplementary Fig. 4a). Significantly more driver mutations were detected by using multi-region WES than that using single-sample sequencing analysis (Wilcoxon rank sum test, *P* = 0.013, Supplementary Fig. 4b). The resulting frequencies of some mutated genes such as *NOTCH1* (33.3%), *PIK3CA* (25.6%), and *KMT2D* (30.8%) were higher compared to that in previously single-tumor studies^6–13^ (Fig. 1c). Most of driver mutations in *TP53*, *PIK3CA*, *ZNF750,* and *BRCA2* were mapped to the trunks of the phylogenetic trees, whereas mutations in *NOTCH1*, *CREBBP*, *PTCH1*, *MET*, *mTOR*, and *AXIN2* almost always occurred in late evolution (Fig. 1c). Particularly, mutations in *ERBB4*, which were previously less reported in ESCC, were observed in five patients (12.8%). Several lines of literatures have linked elevated ERBB signaling to oncogenesis^21^. Its role in ESCC remains elusive^22–24^. In 39-Mseq cohort, we found five novel point mutations of *ERBB4* (Fig. 1d, red line). We integrated previous ESCC studies and found additional 22 of *ERBB4* mutations in the TCGA (1, *n* = 90)^18^, early Chinese (11, *n* = 289)^7,10,16,17,25^, and Japanese (10, *n* = 144)^8^ cohorts. Additionally, we found 13 *ERBB4* mutations in our independent whole-genome sequencing ESCC cohort (*n* = 508, referred as 508-WGS ESCC cohort, Supplementary Fig. 4c). In all, 72.5% of the total 40 mutations occurred in extracellular or kinase domain of ErbB4 (Fig. 1d, Supplementary Fig. 4c), suggesting a potential role of *ERBB4* in ESCC. Three out of 5 novel mutations are within *ERBB4* functional domain; therefore, we constructed the three *ERBB4* mutants within domain and found that over-expression of ErbB4-V391I or ErbB4-P377R increased the basal ErbB4 phosphorylation compared with that of ErbB4 wild type in HEK293T and ESCC cell lines (KYSE150 and KYSE180)^26^ (Fig. 1e, Supplementary Fig. 4d). Moreover, ErbB4-V391I and ErbB4-P377R mutants promoted cell proliferation compared with wild type (Fig. 1e, Supplementary Fig. 4d). These results suggest that *ERBB4* mutations may play an oncogenic role in ESCC and is probably a potential therapeutic target for ESCC treatment.

**Fig. 2:**
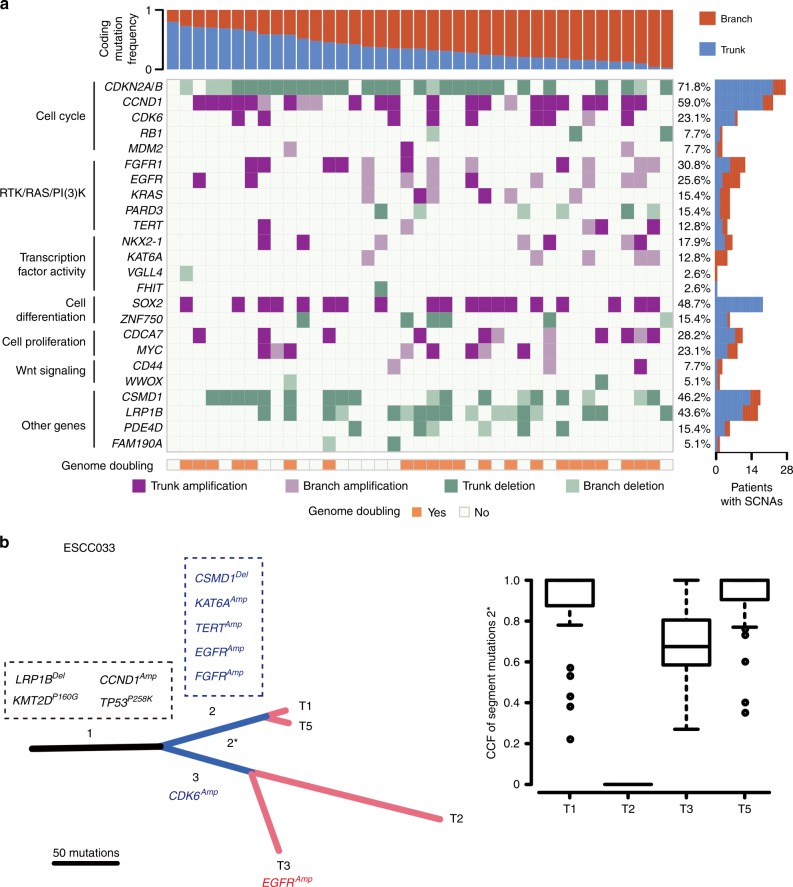
Heterogeneity of amplification/deletion drivers in ESCC. **a** Heat map shows somatic CNAs with different clonal status. The right bar represents the proportion of identified as trunk/branch in ESCC; the bottom panel shows genome doubling status of each patient; the top panel shows the percentages of trunk or branch CNAs. **b** Clonal evolution in case ESCC033. The left panel shows phylogenetic tree based on somatic SNVs. Asterisk segments represent SNVs from intermixed clone; the right panel shows the CCF of segment mutation 2*. Black: trunk; blue: shared branches; red: private branches

In our data, although samples of primary tumors or metastases from the same patient shared most of CNAs, they also differed by a few copy number events that emerged in late stage of tumor evolution. These copy number events distinguish the clones within tumor and often result in amplifications of oncogenes and deletions of tumor suppressors. Here we focused on recurrent CNAs which involved cancer-associated genes in ESCC identified by TCGA. Through the inspection of focal amplifications/deletions of driver genes, we observed the majority of gene amplifications/deletions were trunk alterations in ESCC (Fig. 2a, Supplementary Data 3). However, regarding druggable genes *EGFR* and *FGFR1*, nearly half of amplifications were heterogeneous across samples acquired from the same patient. Thus, blockade to these genes is probably partially effective for these patients. Moreover, when considering druggable gene alterations at pathway level, we found that genes involved in cell cycle regulation, such as *TP53*, *CCND1*, *CDK6*, *RB1*, and *CDKN2A*, were altered in early stage whereas genes involved in *RTK/RAS*/*PI3K* tend to be altered throughout the process of tumor evolution.

Activation of *CDK4/6* pathway may predict response to CDK4/6 inhibitors and provide clinical biomarkers, and the CDK4/6 inhibitor palbociclib showed clinical efficacy in combination with cetuximab (an EGFR inhibitor) in head and neck squamous cell carcinoma^27^. In 39-Mseq cohort, 8 out of 39 patients carried focal amplifications of *CDK6*; of which, amplifications in 7 patients were ubiquitous, highlighting the importance of *CDK6* as a potential druggable target for all tumor cells (TCs) for *CDK6*-amplified patients. The remaining one ESCC033 with high ITH harbored amplifications of *CDK6* and *EGFR* in separate clades (Fig. 2b). Recently study revealed that CDK4/6 blockade enhanced the efficacy of EGFR inhibitor in ESCC^28^. Thus, we speculate that combination of target therapies may be more practical for this patient. These data signify that analyzing sequencing data obtained from multi-sequencing biopsy will be more precise for personalized therapy.

### Novel mutational processes in ESCC

**Fig. 3:**
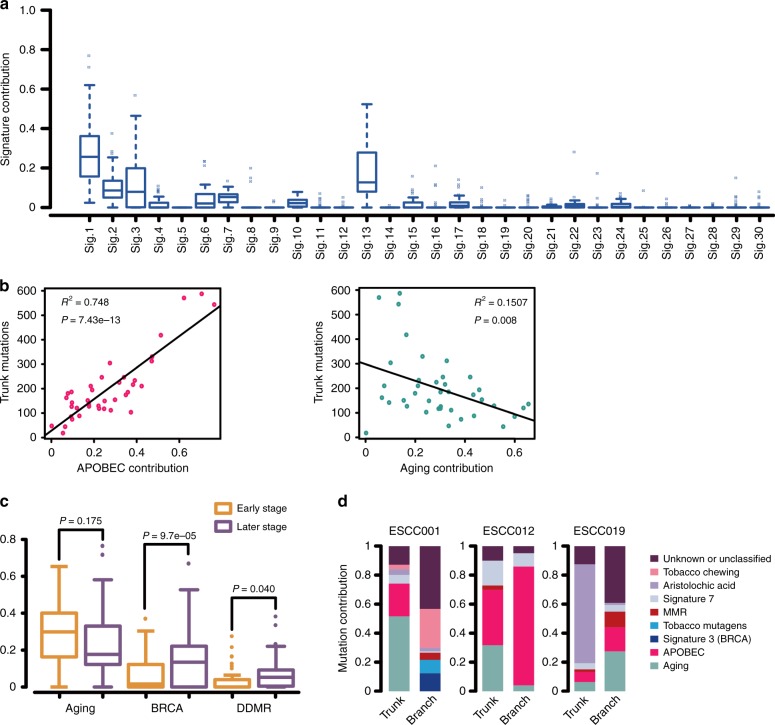
Mutational processes in ESCCs. **a** The relative contribution of each mutational signature across 39 ESCCs. **b** Correlation between trunk mutations and APOBEC or aging signatures. *F*-statistics of the significant test based on linear regression. **c** Box plots in the left show the contribution of aging, BRCA, and DDMR signatures with tumor progression. Brown: early stage; Purple: later stage. **d** Relative contribution of mutational signatures changed between early and later stages in ESCC001, ESCC012, and ESCC019. Eight signatures were found to be important in ESCC, which included signatures associated with tobacco chewing, aristolochic acid, signature 7, MMR, tobacco mutagens, signature 3 (BRCA), APOBEC, and aging. “Unknown or Unclassified” means the other signatures except above

We next analyzed the mutational spectrum of 39 patients to explore the underlying mutational processes operative in ESCC genomes. ESCC012 was separately analyzed due to its hyper-mutated frequency. We then quantified the relevant contributions of 30 COSMIC mutational signatures^29^ to each patient, and identified five dominant signatures, including signature 1 (related to aging), signature 2 and 13 (related to activity of APOBEC family), signature 3 (related to deficient homologous recombination repair), signature 6 (related to mismatch repair deficiency), and signature 22 (related to AA, aristolochic acid) (Fig. 3a). Signature 1, 2, 3, and 13 was each operative with more than 10% contribution in most of patients, whereas signature 22 was detected in only one patient (Supplementary Data 4). Interestingly, the hyper-mutated ESCC012 showed a strong contribution of signature 2 and 13, which accounted for >80% of mutations, suggesting that mutations in ESCC012 may be primarily driven by APOBEC signature (Supplementary Fig. 5 and Supplementary Data 4). Additionally, nine patients exhibited contribution (>0) of signature 29, which has been found in patients with a tobacco chewing habit in other cancers^30^. Particularly, one of these nine patients, ESCC001, showed a relatively higher contribution of signature 29 (~15%). Interestingly, this patient has a long history of smoking in a long-stemmed Chinese pipe with tobacco leaf, which almost has no filter tip and the debris of tobacco leaf is easily sucked into mouth and esophagus, indicating a possible association between signature 29 and tobacco chewing in ESCC. These signatures (signature 3, 22, and 29) were further confirmed in the 508-WGS ESCC cohort (Supplementary Fig. 6a).

To characterize the dynamics of mutational processes during tumor progression, we analyzed the contribution of each mutational process in trunk and branch stage (also referred to early and late stage, respectively). We found that APOBEC and aging processes were predominated in early stage for the majority of 39 patients (Supplementary Fig. 6b). Moreover, the number of trunk mutations significantly correlated with the burden of APOBEC mutations across 39 patients (Fig. 3b), yet this association was not significant for aging mutations. In comparison to early stage, the contributions of *BRCA1/2* and DNA mismatch repair signatures were significantly elevated (*P* = 9.7e-05 and *P* = 0.04, respectively, Student’s *t*-test) while the contribution of aging signature declined in late stage (Fig. 3c, Supplementary Fig. 7). Furthermore, the mutational process was notably varying between early and late stage in some cases (Supplementary Fig. 6b). In case ESCC012, aging and APOBEC signatures were dominant in early stage, but late-arising mutations were primarily driven by APOBEC signature. Another example was ESCC019 that AA signature was enriched in early stage but disappeared in late stage (Fig. 3d, Supplementary Data 5).

### *BRCA1/2* signature as a potential therapeutic target for ESCC

**Fig. 4:**
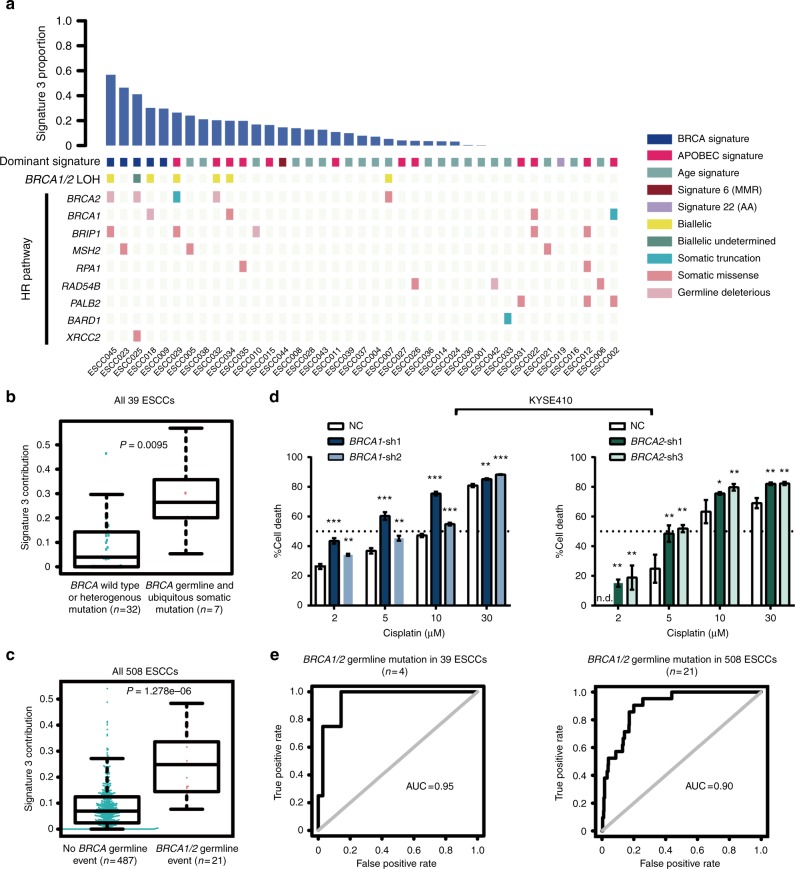
BRCA1/2 signature as a potential therapeutic target for ESCC. **a** Top bar represents the contribution of signature 3 in each ESCC. Colored squares indicate dominant mutational signature and LOH status of *BRCA1/2*. Deleterious germline and somatic mutations in the HR pathway are shown at the bottom of the heat map. **b**, **c** Comparison of signature 3 activity in ESCCs with or without *BRCA1/2* mutation in 39-Mseq cohort (**b**) and additional 508-WGS cohort (**c**). **d** Knockdown of *BRCA1* and *BRCA2* significantly increased sensitivity of KYSE410 cells to cisplatin. ^*^P *<* 0.05, ^**^P *<* 0.01, ^***^P *<* 0.001, n.d. means not detected. Error bars are defined as s.d. Source data are provided as a Source Data file. **e** Receiver operating characteristic (ROC) curves for alterations in *BRCA1/2* germline mutation predicted based on signature 3 levels in 39-Mseq cohort (left) and additional 508-WGS cohort (right)

Several studies have confirmed that signature 3 was strongly associated with *BRCA1/2* mutations in breast, pancreatic, and ovarian cancers^1,2^. In 39-Mseq cohort, four patients with pathogenic germline alterations in *BRCA1/2* genes showed high levels of signature 3, three patients with ubiquitous somatic mutations in *BRCA1/2* also exhibited elevated level of signature 3. By contrast, signature 3 activity was not observed in two patients who harbored branch mutation in *BRCA2*, indicating that patients with late-arising *BRCA1/2* inactivation contributed less signature 3 mutations (Fig. 4a). Loss of heterozygosity (LOH) at the *BRCA1/2* loci was detected in six out of the seven patients with *BRCA1/2* alterations that exhibited high signature 3 level, highlighting the clinical link between the biallelic inactivation of *BRCA1/2* and signature 3 in ESCC (Fig. 4a, b). There were 7 out of 39 patients with a family history of cancer. Of these seven patients, only case ESCC025 had deleterious germline *BRCA1/2* mutations (Fig. 4a), suggesting that family history may not be associated with *BRCA1/2* mutations in ESCC. The association between signature 3 and tumors with other homologous recombination (HR) pathway gene mutations, such as *BRIP1*, *MSH2*, *RAD54B,* and *PALB2*, was less solid (Fig. 4a). Furthermore, a number of patients without HR pathway defect were in the top quartile of signature 3 activity, suggesting that other events may contribute to signature 3. Notably, these results were further verified in the 508-WGS ESCC cohort, in which elevated signature 3 contribution was observed in 20 out of 21 ESCCs with germline *BRCA1/2* variant but not in tumors harboring somatic mutations in other HR pathway genes (Fig. 4c, Supplementary Fig. 8, Supplementary Fig. 9a). Since the specific sensitivity of tumors with *BRCA1/2* mutations to platinum therapy was described in breast cancer and ovarian cancer^31,32^, we expected the effects to be identical in ESCC. As a result, *BRCA1/2* knockdown dramatically increased sensitivity to cisplatin in KYSE410 and ZEC-014-1 cells (Fig. 4d, Supplementary Fig. 9b, c), indicating a potential novel therapeutic strategy to ESCC patients with *BRCA1/2* mutations.

Although *BRCA1/2* signature was validated to predict *BRCA1/2* deficiency with high sensitivity and specificity in breast cancer^33^, its predictive value in ESCC remains unclear. We therefore empirically estimated the area under the receiver operating characteristic curve (AUC) between signature 3 contribution and *BRCA1/2* mutations. The calculated AUC was 0.84 for detecting biallelic inactivation in 39 patients (Supplementary Fig. 9d). The AUC value was increased to 0.88 when included ESCC025 whose LOH status was undetermined and was increased to 0.95 when calculated based on *BRCA1/2* germline events (Fig. 4e, Supplementary Fig. 9e). Signature 3 activity was reapplied on the 508-WGS ESCCs with an excellent AUC of 0.90 when considered only pathogenic germline mutations (Fig. 4e). Hence, signature 3 may serve as a potential predictor of germline *BRCA1*/*BRCA2* deficiency in ESCC.

### Evolutionary patterns within ESCC

**Fig. 5:**
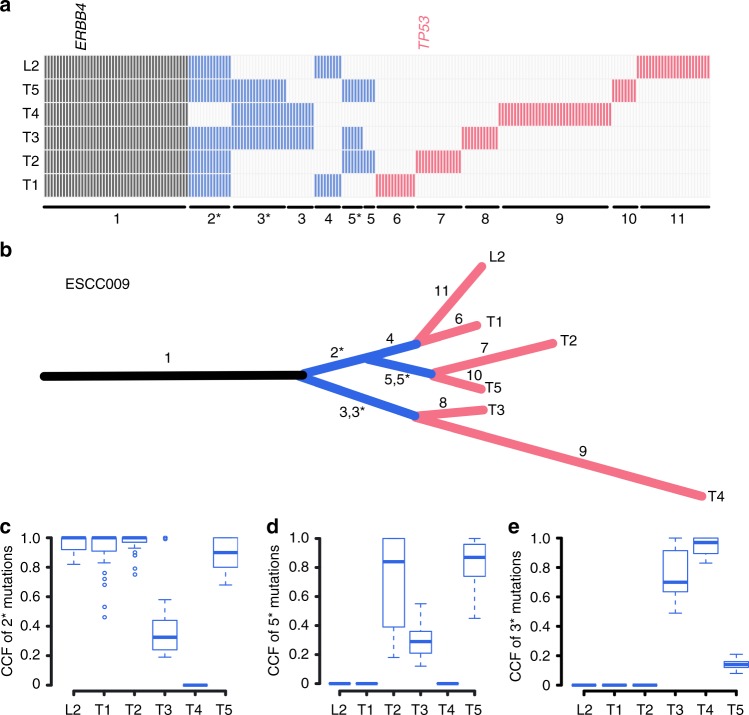
Evolutionary patterns within ESCC009. **a** The distribution of non-silent mutations in different regions of case ESCC009. **b** Phylogenetic tree of ESCC009 based on somatic SNVs. Black: trunk; blue: shared branches; red: private branches. **c–e** The CCF of somatic SNVs in specific shared branches

To investigate the universal evolutionary patterns within ESCC, we constructed the multi-region tree of tumors based on somatic single-nucleotide variants (SNVs) and further used CNA data to support the final phylogenetic trees (Methods, Supplementary Figs. 10, 11). Intermixed clones were obviously observed in 5 out of 39 (12.8%) patients (Figs. 2b, 5, Supplementary Fig. 12). The intermixing pattern was very complex in some patients. For example, for ESCC009, we found two main clades involving many subclones during diversification (Fig. 5). Region T3 displayed both SNVs acquired in the evolutionary history of clade comprising primary tumors T1, T2, T5, and lymph-node metastasis (segment 2*, Fig. 5b, c) as well as SNVs private to sub-clade of T2 and T5 (segment 5*, Fig. 5b, d). On the other hand, T5 also encompassed SNVs from the clade 2 consisting of T3 and T4 (segment 3*, Fig. 5b, e). In case of ESCC033 (Fig. 2b), T3 harbored both SNVs from clade of T1 and T5 (segments 2*) and SNVs private to T2 (segment 3*). Intermixed samples were also observed in patients ESCC035, ESCC026, and ESCC043 (Supplementary Fig. 12). Further, to explore how subclones evolved after transformation, we applied the neutral evolutionary model^34^ to 69 samples with at least 50% tumor content and found that 61% tumors follow neutral evolution (Methods, Supplementary Fig. 13 and Supplementary Data 6).

**Fig. 6:**
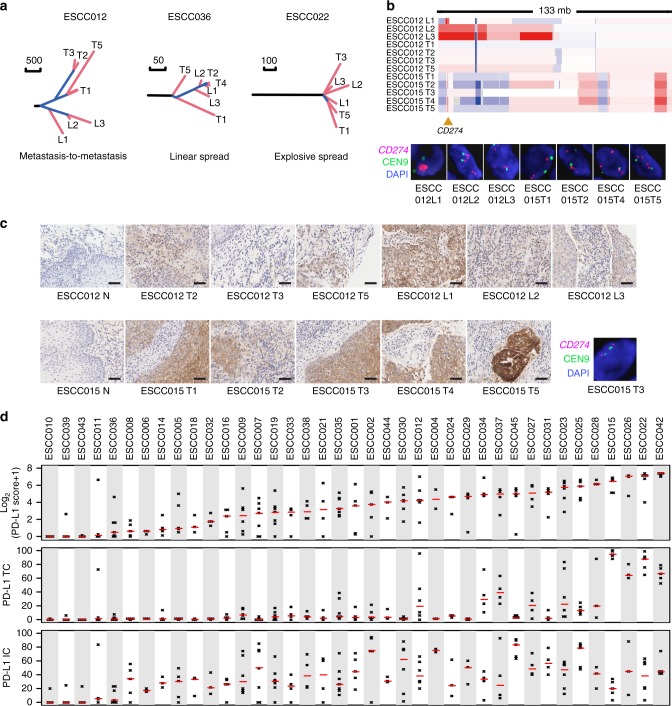
ITH of amplification and protein expression of *CD274* (PD-L1) in ESCC. **a** Representative phylogeny for metastasis patterns of ESCC. **b** ITH of amplification in case ESCC012 and ESCC015. The bottom panel displays FISH validation of *CD274* amplifications. **c** IHC staining of PD-L1 on samples from patients ESCC012 and ESCC015. Right bottom shows the absence of *CD274* amplification using FISH analysis in ESCC015 T3. Scale bar: 50 μm. **d** PD-L1 protein expression in TC, IC, and tumor tissue

To investigate the evolutionary pattern of lymph-node metastasis in ESCC, 10 patients with metastatic lymph nodes were inspected. Interestingly, metastases in each patient were clonally related with primary tumor and originated from cells disseminated at various stage of the disease. A representative phylogeny for ESCC012 is illustrated in Fig. 6a. All the metastases clustered on one clade and descended from what refer to as the “metastatic precursor”. All the samples from primary tumor clustered on the other clade. We compared cancer cell fraction (CCF) for each branch mutation, but found no intermixed mutations between these two clades. Therefore, metastases probably arose via a monoclonal seeding to an initial “metastatic precursor” from the primary tumor in this patient, indicating that further dissemination among metastases probably occurred by metastasis-to-metastasis dissemination in this patient. ESCC036 showed a linear spread pattern: one subclone from primary tumor subsequently seeded all the metastases L1–3 (Fig. 6a). In contrast, ESCC022 probably underwent an explosive metastasis as metastatic subclones separately seeded metastases L1–3 in early stage after carcinoma transformation (Fig. 6a). Altogether, these data unveil diverse patterns of metastatic spread in ESCC.

Parallel evolution converging on the same genes in different subclones could increase ITH. The mirrored subclonal allelic imbalance (MSAI, Methods) events were observed in 46% of 39 patients ranging from focal CNAs to arm-level changes (Supplementary Data 7). In addition, distinct types of amplifications with different boundaries and copy number ratios were found to occur on the same gene in different subclones, including driver genes *EGFR*, *CCND1*, and *MYC* (Supplementary Fig. 14). Intriguingly, amplification of *PD-L1* (*CD274*), an important biomarker for immunotherapy, was heterogeneous in patients ESCC015 and ESCC012. Of eight sequenced regional samples in ESCC012, three lymph nodes with metastatic TCs harbored *PD-L1* amplifications (≥4 copies). L1 harbors focal and high-level amplification of *PD-L1* while L2 and L3 have broad amplification of *PD-L1* (>20 Mbp) (Fig. 6b). In ESCC015, except for regional sample T3 that diverged from other regions in early stage of tumor evolution, the other four regional samples harbored focal *PD-L1* amplification. These results were further validated by fluorescence in situ hybridization (FISH) analysis (Fig. 6b, c). Parallel evolution also involved mutations of driver genes, such as *NOTCH1*, *TP53,* and *PIK3CA* mutations, of which parallel *NOTCH1* mutations were found in patients ESCC015 and ESCC043 (Supplementary Data 2).

### Heterogeneous expression of PD-L1

To examine whether *CD274* amplifications are associated with PD-L1 protein level, we performed immunohistochemical (IHC) analysis to evaluate PD-L1 expression. The seven *CD274* amplified samples containing TCs and immune cell (IC) infiltrates were positively stained for PD-L1 (Fig. 6c, d). Of which, five focal amplified samples demonstrated massive expression in TCs (Fig. 6c, d). Meanwhile, we searched for focal *CD274-* amplified ESCCs from TCGA (90 ESCCs available) and found that two high-level amplified tumors exhibited high transcript levels of *CD274* and IC markers (Supplementary Fig. 15). These data implicate that focal *CD274* amplification may be associated with PD-L1 expression in ESCC. Moreover, the genomic heterogeneity of *CD274* led to differential expression of PD-L1 in regions for ESCC012 but caused no significant changes for ESCC015, whose T3 region showing the absence of *CD274* amplification (Fig. 6b upper, c right bottom) but the presence of PD-L1 expression, indicating that PD-L1 expression may be regulated by other mechanisms, simultaneously.

Given that the temporal and spatial heterogeneity of PD-L1 expression was not previously implicated in ESCC, we further compared the PD-L1 protein level among intra- and inter-patient. Of 185 samples, 69% was found to be positive for PD-L1 staining. Moreover, PD-L1 expression in TCs was detected in 70% of samples, and approximately 90% exhibited PD-L1 expression in tumor-infiltrating ICs. For 39 adjacent normal tissues, ICs from seven patients were positive for PD-L1 staining. We found that 37/39 patients had at least one PD-L1-positive sample; 28/39 patients had two or more PD-L1-positive samples; only 14/39 (35.9%) patients showed PD-L1 protein expression in all samples (Fig. 6d). Collectively, these findings reveal that PD-L1 expression may be highly heterogeneous within different spatial regions in ESCC.

### ITH of T cell immune repertoire

**Fig. 7:**
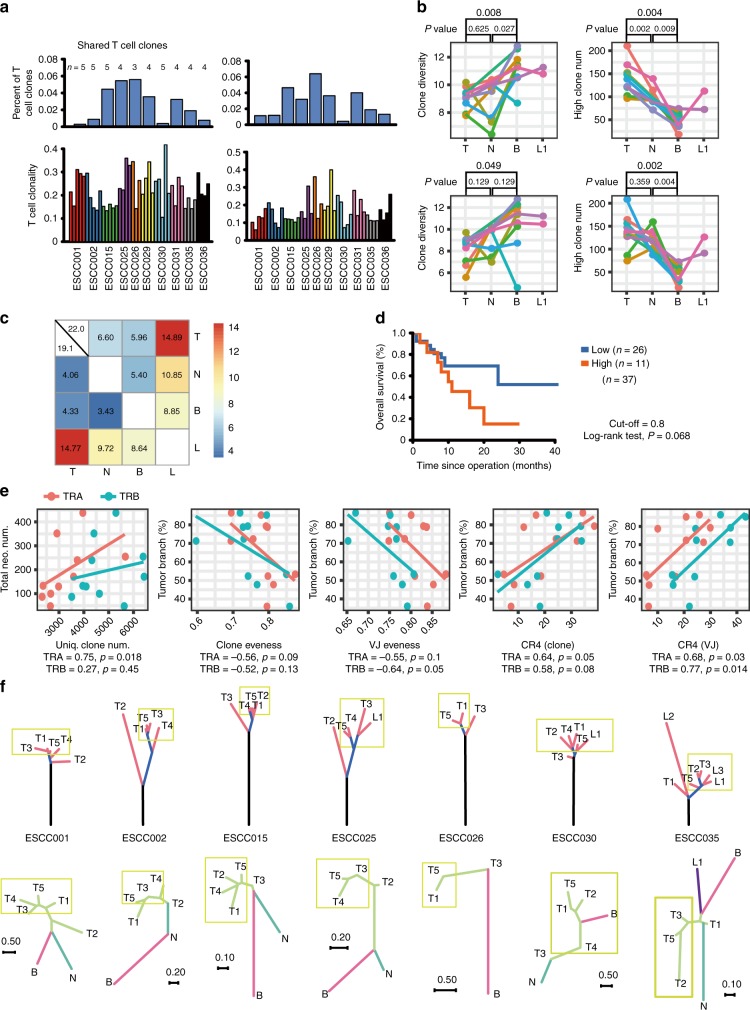
Analysis and statistics of TCR repertoires in localized ESCC. **a** The percentage of shared TCRs in all primary tumor regions (top panel) and T cell clonality in each primary tumor region (bottom panel). The left panel shows statistics of TRA and the right panel is for TRB. **b** The overall clone diversity (Shannon index, left panel) and high-frequent clone number (freq. > 0.01%, right panel) of TRA (top panel) and TRB (bottom panel) for four sorts of samples. T primary tumor sample, N normal sample,B blood sample, L lymphoma metastasis. **c** The median overlapped rate between any two types of samples shown by heat map. The color in the heat map represents the values of Jaccard indices (range: 0–100). The left bottom corner shows result of TRA repertoires and the right top corner shows that of TRB repertoires. **d** Kaplan–Meier survival curves displaying survival outcomes of ESCCs with distinct neoantigen heterogeneity. **e** Correlation of T cell repertoire with branch neoantigens. Spearman correlation between the median of unique clone number in primary tumors and total neoantigen number (left); Spearman correlation between the percentage of predicted branch neoantigen and the median of clone evenness, V–J pairing’ evenness, CR4 of clones, and CR4 of V–J gene pairings in primary tumors. **f** Comparison of TCR tree and genetic tree in seven patients. The upper panel displays the cancer evolutionary trees based on somatic SNVs, and the trunk, branch, and shared branch are colored in dark, blue, and red, respectively; the bottom panel shows the unrooted neighbor-joining tree based on TCR, and the trees are colored by tissue types (lake blue: normal; green: regional tumor; pink: blood; purple: metastatic lymph node). Yellow rectangles highlight regional tumors from the same clade that have more similarity in TCR tree

To further investigate the heterogeneity of immune microenvironment within ESCC patients, we analyzed the data from 10 patients consisting of 64 samples subjected to immune repertoire sequencing (Supplementary Fig. 1). T cell clonality was generally comparable for both alpha and beta chain of T cell receptor (TRA and TRB) between different patients. However, within intra-patients, we observed variations of overall TCR repertoire, including the T cell clonality, high-frequent clones, and V–J gene pairing diversity (Fig. 7a and Supplementary Fig. 16a). The shared T cell clones were very few within primary tumor samples and showed great diversity in different patients (Fig. 7a). Few shared top clones were also observed within patients (Supplementary Fig. 17), and these data implied the potential differences of the immunogenicity. Further, TCR diversities were higher in blood compared to that of normal and tumor tissues, while there was no difference between normal and tumor tissues (Fig. 7b, Supplementary Fig. 16b). The number of high-frequency clones (>0.1%) was elevated in primary tumors compared to that in blood and normal tissues for both TRA and TRB (Fig. 7b), which was probably due to the arising neoantigens. Moreover, we also utilized Jaccard index to quantify the similarity and overlap of the T cell repertoire among normal, tumor tissues, and lymphatic metastases (Fig. 7c and Supplementary Fig. 18). We found that the relationship among primary tumor samples is closest, and followed by between primary tumors and lymphatic metastases (Fig. 7c). The relatively more similarity of TCR repertoire among primary tumors and lymphatic metastases might result from the stimulation of cancer cells. Similar pattern was observed for the correlation of V gene, J gene, and VJ gene pairings (Supplementary Fig. 19).

To identify potential biological and clinical relevance that was associated with TCR diversity, we analyzed clinical information and neoantigen, especially branch neoantigens that were more likely to influence the heterogeneity of TCR repertoire. The high proportion of branch neoantigens was associated with short overall survival in a marginal significance, which was probably due to the insufficient sample size (Fig. 7d). We found that the total neoantigen number was associated with unique clone number of TRA (Fig. 7e). In addition, the percentage of branch neoantigens was negatively correlated with clone evenness and V–J pairings’ evenness. We also found a positive correlation between the branch neoantigen and high-frequent clones or V–J pairings (Fig. 7e). These findings suggest that branch neoantigens may relate with the TCR’s overall diversity and some expanded TCR clones probably respond to the increase of variety of TCs.

To further visualize the similarity of samples for TCR repertoire in each patient, we construct the phylogenetic tree based on the neighbor-joining method using distance metrics proposed by *Harlan*^35^. The repertoire of different regions of the same primary tumor is more similar than for tumor vs. blood or normal tissue, and the blood and normal tissue always located in the adjacent evolutionary tree branches (Supplementary Fig. 20). We further compared the topology of regional tumors from TCR tree to that from genetic tree. Notably, we found that, in 7 out of 10 patients, regional tumors belonging to the same clade from genetic tree tend to have more similarity in TCR trees compared to those from other clades (Fig. 7f). These results suggested that the ITH of TCR repertoire within ESCC was driven to a large extent by genetic heterogeneity in different tumor regions.

## Discussion

Our study provides the first report of mutational signature 3 and its association with ubiquitous somatic or deleterious *BRCA1/2* germline variants in ESCC. Moreover, *BRCA1/2* knockdown ESCC cells were more sensitive to platinum treatment. These results are probably helpful for treatment of ESCC patients with *BRCA* mutation, especially for inoperable ones. Signature 3 demonstrated its capability to be an actionable predictor in pancreatic and advanced breast cancer^1,2^. In our data, we found that some patients (sporadic or familial) with high-level signature 3 had no *BRCA1/2* mutations that were probably owing to epigenetic changes or other events. Thus, signature 3 may provide an indication of sensitivity to platinum therapy in ESCC. Nevertheless, only limited clinical information was available for ESCC patients and most of them were subjected to surgery prior to chemotherapy which restricts our opportunities for exploring potential association of signature 3 with disease progression and outcome following platinum therapy and PARP inhibitors. Further analyses, involving larger ESCC cohorts with complete clinical data, specifically with treatment information on platinum or PARP inhibitor, may help to elucidate the connection between signature 3 and clinical response in ESCC.

Through multi-region sequencing, we constructed phylogenetic tree for each ESCC patient that mapped driver gene mutations in order of time. Our results redefined the mutational landscape at the patient level. The high mutation rate (>12.8%) of *ERBB4* was identified, and the oncogenic role of its mutants was confirmed by experiments. Moreover, at CNA level, we found high ITH of druggable gene *EGFR* and *FGFR1* in approximately 50% *FGFR1*- and *EGFR*-amplified cases, indicating that the mutation status of these two targets from single-tumor sequencing may underestimate their ITH. Importantly, focal amplification of PD-L1 was associated with massive PD-L1 expression of TCs, suggesting that it may act as a useful predictor for PD-L1 blockade. Collectively, these data suggest that multi-regional sampling is necessary for driver gene identification and biomarker prediction in the future.

In addition to the finding, high proportion of branch neoantigens was associated with overall short survival. We also observed that, within primary tumor, the relationship of spatial TCR repertoire was correlated with genomic ITH to some extent, suggesting the ITH that sourced from genome evolution may have clinical relevance in ESCC by affecting the changes of the tumor immune microenvironment.

A small proportion of patients with low expression of PD-L1 showed stable responses to PD-L1 blockade in other types of cancer^36^. We demonstrated the heterogeneity in TCR landscape and PD-L1 expression within ESCC patients, indicating that the status of PD-L1 expression may be incorrectly assigned due to the spatial heterogeneity. PD-1 blockade predominantly activates T cells within tumor, and CTLA-4 blockade could help T cell activation and trafficking^37^. Therefore, combination of these two agents may help T cells travel not only to the tumor, but within the tumor, and ultimately make the immune infiltrates more consistent within the tumor and generate more effective anti-tumor responses. Although several pre-clinical studies and preliminary trials of combination therapy with anti-PD-1/PD-L1 plus anti-CTLA-4 immune-checkpoint inhibitors have documented considerable advantages in survival over single-agent immune-therapy^38^, toxicity may presumably be a major issue. The safety statistics from clinical trials showed that adverse events related with treatment were observed in larger proportion of patients receiving the concurrent regimen compared to those receiving single-agent immunotherapy only^39,40^.

In this study, we also attempted to explore the evolutionary pattern of ESCC, especially from the stage of carcinoma initiation to tumor growth or lymph-node metastases. Further, three patterns of metastatic seeding, metastasis-to-metastasis, linear and explosive, were observed in our data, suggesting that seeding clones of different lineages could occur in a punctuated pattern instead of a gradual evolution. However, the specific features related to primary ESCC’s favoring one metastasis model over others are still unclear. Further studies in larger patient cohorts are needed to accurately quantify the abundance of each metastasis manner and its association with clinical metrics and treatment, which would serve to improve the clinical management of metastatic ESCC patients. Especially, since distinct metastasis evolves independently, biopsies from a single metastatic site may be insufficient for clinical decision for ESCC patients predominated with explosive manner.

## Methods

### Sample selection

Samples obtained from 39 patients recruited from Shanxi province, China were used in this study (Fig. 1a). All patients signed their own informed consent and all samples were obtained before treatment according to the guidelines of the local ethical committees. All ESCC patients were staged according to the Cancer Staging Standards of the American Joint Committee on Cancer (seventh edition, 2010). Primary tumor samples and matched adjacent histologically normal tissues were obtained from 39 ESCC individuals and frozen into liquid nitrogen within 30 min post-surgery. The length, width, and depth of tissue samples were measured immediately after dissection in operation, and then five spatially isolated tumor specimens were obtained per patient with each primary tumor at least 0.5 cm away from the others. Meanwhile, we collected 21 metastatic lymph nodes (1–3 samples per patient, from 10 patients) and peripheral blood mononuclear cells (PBMCs, 1 sample per patient, from 10 patients). The PBMCs were isolated from whole blood immediately after blood drawing by using Human Peripheral Blood Lymphocyte Isolation Kit (LTS1077, Tbdscience, Tianjin, China) according to the manufacturer’s protocol. All samples were stocked in a −80 °C freezer until DNA extraction. After quality control of sequencing data, we removed those samples with low-quality data and performed analyses by using the samples with good quality data, which include 39 normal esophageal tissues and 164 regional tumors from 39 patients, 21 metastatic lymph nodes samples and PBMCs from 10 patients. Detailed clinical characteristics of 39 ESCC individuals are provided in Supplementary Data 1.

For the 508-WGS cohort, we performed deep WGS of microdissected tumor tissues and matched adjacent non-cancerous specimens from 508 ESCC patients with detailed medical records and survival data. All tumors were therapy naïve. Paired-end sequencing was performed using an Illumina HiSeq system following Illumina’s instructions in WuXi NextCODE at Shanghai, China. The mean coverage of sequencing of the tumor tissues was 98× and 44× in matched non-tumor samples.

All research subjects provided written informed consent to participate in this study. The ethical committees of the Shanxi Medical University approved the study.

### Whole-exome sequencing

Genomic DNAs were extracted from regional tumors, metastatic lymph nodes, and matched adjacent normal tissues. WES libraries constructed as follows: a total amount of 0.6 µg genomic DNA per sample was used as input material for the DNA sample preparation. Sequencing libraries were generated using Agilent SureSelect Human All Exon V6 kit (Agilent Technologies, CA, USA) following the manufacturer’s recommendations and index codes were added to each sample. Briefly, fragmentation was carried out by the hydrodynamic shearing system (Covaris, Massachusetts, USA) to generate 180–280 bp fragments. DNA fragments with ligated adapter molecules were selectively enriched by PCR, and then exons of genes were captured with a biotin-labeled probe. Captured libraries were enriched by PCR to add index tags to prepare for sequencing. Products were purified using the AMPure XP system (Beckman Coulter, Beverly, USA) and quantified using the Agilent high sensitivity DNA assay on the Agilent Bioanalyzer 2100 system. Then sequencing was performed on an Illumina HiSeq X-Ten sequencer with 150-bp paired-end reads.

High-quality reads were aligned to NCBI human reference genome (hg19); duplicate marking was performed using Picard, achieving an average sequencing depth of 290× and 187× for tumors and normal samples, respectively. For mutation validation, we randomly chose three samples (ESCC012 T5, ESCC014 T1, ESCC035 L2) with high mutation rate (2 standard deviations larger than the mean value of the regions in the same patient), re-constructed WES library, and performed the same sequencing and mutation-calling strategy.

### TCR repertoire sequencing

Due to the limited blood samples available, we only performed TCR repertoire sequencing on 10 patients (ESCC001, ESCC002, ESCC015, ESCC025, ESCC026, ESCC029, ESCC030, ESCC031, ESCC035, and ESCC036). gDNA of 64 samples including the adjacent normal esophageal tissues, the regional tumors, the metastatic lymph nodes, and the PBMCs were extracted, and subjected to amplify TCR alpha and beta repertoire using multiple PCR^41^. The V gene segments, J gene segments, and the whole complementarity determining region 3 (CDR3) were captured by PCR reactions^41^. Amplification products were sequenced by xTen with 150-bp paired-end reads. Raw sequencing data were processed and filtered using IMonitor^42^. Clean sequences were aligned to V and J germline alleles (IMGT database, www.imgt.org), and V and J gene segments were assigned for each clone. Meanwhile, the sequencing errors were corrected in terms of sequencing qualities and low-frequent sequences (≤2 in one million) were filtered out.

### Removing samples with low tumor cellularity

Samples with low tumor content often have ambiguous copy number changes and mutations of very low variant allele frequency. Thus, the detected somatic mutations and CNAs from these low purity samples are inaccurate and insensitive for follow-up analyses. ESCC is a disease with highly unstable genome, and all of ESCCs that were previously reported have a large number of copy number changes and mutations. Here, we manually inspected copy number profile for each sample and removed samples harboring no or ambiguous copy number changes. In addition, we also ruled out samples contaminating other human DNAs that might be cross-contamination during sequencing.

### Somatic mutations calling

Somatic SNVs were called by MutTect^43^ with default parameters based on paired alignment files (tumor and normal), and filtered these SNVs with supported reads ≥4 (≤2) and coverage ≥14 (≥10) in tumors (normal). The resulting SNVs were filtered for false positives using fpFilter Perl script (https://github.com/ckandoth/variant-filter). Somatic indels (insertions or deletions) were called by Pindel with supported reads ≥6, coverage ≥20, and VAF ≥ 0.1. We further filtered false positives by manual checking for indels. Finally, those filtered high-quality somatic variants were annotated by an Oncotator^44^. One tumor was considered as hyper-mutated as it had ≥10× median mutation burden.

### Mutation signature analysis

To estimate the contribution of 30 mutational signatures documented by the COSMIC, we applied R package Mutational Patterns (https://github.com/UMCUGenetics/Mutational Patterns) to each sample. Signature with the highest proportion of the contribution was considered as the dominant signature for each sample.

### Germline variants calling

Germline variants were called by Platypus (v0.8.1)^45^ with default parameter. Variants among 25 DNA repair genes (*BRCA1*, *BRCA2*, *MRE11A*, *NBN*, *RAD50*, *RAD51C*, *RAD51B*, *RAD52*, *XRCC2*, *BLM*, *EME1*, *RPA1*, *POLD1*, *TOP3A*, *RAD54B*, *TOP3B*, *BARD1*, *BRIP1*, *MLH1*, *MSH2*, *PALB2*, *CHEK2*, *FAM175A*, *MSH6*, *RAD51D*) were extracted from the call set and filtered with at least 30× coverage and variant allele frequency (VAF) >8%. Common variants (with a minor allele frequency >1% in either the Exome Variant Server (EVS; http://evs.gs.washington.edu/EVS/) or the ExAC normal population cohort)^46^ were removed. Germline deleterious mutations were defined as either truncating alteration (nonsense, frameshift, or splice site mutations) or missense mutation annotated be pathogenic in the ClinVar database (https://www.ncbi.nlm.nih.gov/clinvar/).

### Copy number calling and CCF analysis

Copy number profiling was estimated by using ReCapSeg, which is a part of GATK (v4)^47^. Heterozygous single-nucleotide polymorphisms (SNPs) were called using Platypus (v0.8.1)^45^ with default parameters apart from the genIndels flag set to FALSE. The LogR of segments and VAF values of heterozygous mutations were then processed with AllelicCapseg (http://archive.broadinstitute.org/cancer/cga/acsbeta) to generate allelic segmentation copy number data for all samples.

Tumor ploidy and purity were determined on the local copy number of each segment and the allele frequency of each somatic SNV using ABSOLUTE^48^. CCF was measured for each mutation. Mutations were classified as subclonal if the upper bound of CCF 95% confidence interval was less than 1. To determine LOH at *BRCA1/2* loci, absolute copy numbers were estimated from the genome-wide profile. A gene was labeled as having LOH when one allele was clonally deleted and the other allele was not clonally deleted. Tumor samples with loss of alternate allele in case of LOH were defined as those with a variant read frequency of >0.7^49^.

### Identification of putative driver mutations

We first identified a list of putative cancer driver genes (*n* = 720) comprised of all genes reported in previous ESCC sequencing studies^7,8,10,11,13,18,25^, the COSMIC cancer gene census^50^ (v82) and pan-cancer analysis^51^. Next, non-silent variants that were located within these genes were evaluated if they met one of the following criteria: (i) either an exact match or at least three mutations located within 15 bp of the variant were found in COSMIC, and (ii) if the gene was annotated as recessive by COSMIC and the variant was deemed to be deleterious, including stop-gain, frameshift, and splicing mutation, and had a SIFT score <0.05 or a PolyPhen score >0.995 (refs. ^52,53^).

### Detection of mirrored subclonal allelic imbalance

We applied Jamal-Hanjani’s method to detect MSAI from multi-region sequencing data^20^. Briefly, breakpoints from each patient tumor sample’s segmentation profile were combined to create a single patient-specific consensus segmentation profile. Then for each consensus segment with allelic imbalance, two log-truncated normal distributions were created with their μ set as the theoretical B-allele fraction (BAF) corresponding to major copy number and minor copy number. The SNPs were then assigned a log likelihood of belonging to either major or minor copy number using these distributions. The probabilities of a reversal having occurred for each SNP’s BAF were also calculated. Finally, these values were compared using one-tailed *t*-test to determine whether the log likelihood ratio values of these SNP BAFs constituting an MSAI event were significantly different from zero.

### Neutral evolution model

Sixty-nine out of 185 samples (64 regional tumors and 21 metastatic lymph nodes) had >50% tumor content to allow the study of evolutionary processes. Due to the well-known linear and branched evolution in ESCC and other human cancers, we mainly focused on the neutral evolutionary model. Neutral evolution was estimated based on a parameter-free model described by Marc J. Williams et al.^34^. As the author recommended, high purity (>0.5) samples with at least 12 SNVs with a VAF within the boundary of (0.12, 0.24) were included. A linear model was fitted using Eq. (1):

1 $$M\left( f \right) = \frac{\mu }{\beta }\left( {\frac{1}{f} - \frac{f}{{f_{{\mathrm{{max}}}}}}} \right),$$

where *M*(*f*) represents the cumulative number of mutations per frequency, *f* means VAF, *f*_max_ means expected VAF of clonal mutations, and *μ*/*β* means mutation rate per effective cell division, which corresponds to the slope of *M*(*f*). An *R*^2^ ≥ 0.98 indicates neutral evolution. For samples with *R*^2^ < 0.98, we applied a purity corrected model with Eq. (2):

2 $$M\left( f \right) = \frac{\mu }{{\beta (1 + \varepsilon )}}\left( {\frac{1}{f} - \frac{f}{{f_{{\mathrm{{max}}}}}}} \right),$$

where *ε* represents the normal contamination. Samples with *R*^2^_adjust_ ≥ 0.98 were then called neutral evolution.

### Phylogenetic tree construction

To investigate mutational heterogeneity, we extracted read information of all tumor samples from one patient by using bam-readcount (0.5.1; https://github.com/genome/bam-readcount) for each SNV. Mutations with VAF ≥ 2% were allowed for subsequent analysis that could identify low frequency variants. The VAFs of all tumor samples from each patient were converted into a binary table that was utilized by Discrete Character Parsimony, implemented in PHYLogeny Inference Package (http://evolution.genetics.washington.edu/phylip.html), to produce the phylogenetic trees. Based on the branch/trunk lengths calculated from mutations counts, the raw phylogenetic trees were constructed manually. Additionally, we also used branch-specific CNAs available to get the final trees. In return, phylogenetic trees were used to adjust the focal driver CNAs status in some regional samples. For instance, *CDKN2A* deletion was absent in T1 and T5 samples from ESCC015 patient (Supplementary Data 2); however, it is not consistent with phylogenetic tree shown in Supplementary Figs. 10, 11. Similar observations exist in several patients (Supplementary Data 2) that were revised according to the polygenetic trees.

### Whole-genome sequencing of 508 ESCC patients

High-quality total DNA from 10 mg microdissected tissues was extracted by the Maxwell 16 Tissue DNA Purification Kit (Promega). The integrity and concentration of total DNA were determined by agarose electrophoresis and Qubit 2.0 fluorometer dsDNA HS Assay (Thermo Fisher Scientific). Approximately 300 ng high-quality DNA sample (OD_260/280_ = 1.8 ~ 2.0) was sheared with Covaris S220 Sonicator (Covaris) to ~350 bp. Fragmented DNA was purified using Sample Purification Beads (Illumina). Adapter-ligated libraries were prepared with the TruSeq Nano DNA Sample Prep Kits (Illumina). After library construction, Qubit 2.0 fluorometer dsDNA HS Assay (Thermo Fisher Scientific) was used to quantify concentration of the libraries while the size distribution was analyzed by an Agilent BioAnalyzer 2100 (Agilent). The Illumina cBOT cluster generation system with HiSeq PE Cluster Kits (Illumina) was used to generate clusters. Paired-end sequencing was performed using an Illumina HiSeq system following the Illumina’s instructions for 2 × 150 paired-end sequencing in WuXi NextCODE at Shanghai, China.

### TCR repertoire analysis

We only used productive clones for further analysis. The analyses mainly include three parts. Firstly, we calculated overall diversity on both clones and V–J parings as well as the high-frequent clone and V–J pairings, which include clone clonality^54^, unique clone number, clones’ Shannon index^42^, high-frequent (>0.01%) clone number, concentration ratio 4 (CR4, the sum frequency of top 4 clones or V–J pairings) and so on. The evenness of clone repertoire or V–J parings was calculated by Peilou, we computed by Eq. (3),

3 $${\mathrm{{Evenness}}} = \frac{{{\mathrm{{Shannon}}\,{\mathrm{Index}}}}}{{{\mathrm{{log}}}_2\left( {{\mathrm{{unique}}}\,{\mathrm{{clone}}}\,{\mathrm{{number}}}} \right)}}.$$

In this step, to normalize the sequence data, we selected randomly one million sequences to calculate these indexes. In the second step, we used the Jaccard Index to evaluate the TCR repertoires’ overlapped rate between two samples. Finally, for each patient, we calculated pairwise distances to generate a distance metric from the overlapped rates (Jaccard Index). We used the same weight for both TRA and TRB repertoires. Formula (4) of distance listed as follows:

4 $${\mathrm{{Distance}}} = 0.5 \left( {\frac{1}{{{\mathrm{{Jaccard}}}\,{\mathrm{{index}}}\,({\mathrm{{TRA}}})}} - 1} \right) + 0.5 \left( {\frac{1}{{{\mathrm{{Jaccard}}}\,{\mathrm{{index}}}\,({\mathrm{{TRB}}})}} - 1} \right).$$

### Neighbor-joining tree construction based on TCR data

To build up neighbor-joining tree for 10 patients with repertoire sequencing, we calculated pairwise distances to generate a distance metric from overlap metric, which was reported in previously published paper^35^. We used the same weight for both TRA and TRB repertoires. The formula (5a and 5b) of distance listed as follows:

5a $${\mathrm{overlap}} = \frac{{\mathop {\sum }\nolimits_{i = 1}^n a_i + b_i}}{{\mathop {\sum } A + {\sum} B }},$$

5b $${\mathrm{{Distance}}} = 0.5 \left( {\frac{1}{{{\mathrm{{overlap}}}\, ({\mathrm{{TRA}}})}} - 1} \right) + 0.5 \left( {\frac{1}{{{\mathrm{{overlap}}}\, ({\mathrm{{TRB}}})}} - 1} \right),$$

where *a*_*i*_ and *b*_*i*_ were the read counts of shared CDR3 *i* in two samples, and ∑*A* and ∑*B* were the total sequence amounts for the two samples, respectively. We utilized the distance metric to construct a neighbor-joining tree using MEGA7 software^55^.

### Neoantigen prediction

To predict patient-specific neoantigens, we firstly determined the 4-digit HLA type using POLYSOLVER^56^ for all tumor regions of each patient. Then, we used non-silent mutations to generate a comprehensive list of peptides ranging 8–11 amino acids in length with the mutated residues represented in each position. The MHC class I-binding affinity of mutant peptide and its corresponding wild-type peptide to the patients’ germline HLA alleles was estimated with netMHCpan-3.0 (ref. ^57^). Finally, we defined candidate neoantigens as those with a predicted binding affinity lower than 500 nM.

### Cell culture

HEK293T was cultured in Dulbecco’s modified Eagle's medium (DMEM) containing 10% fetal bovine serum (FBS), 50 U mL^−1^ penicillin and 50 U mL^−1^ streptomycin. ESCC cell lines (KYSE180, KYSE150, KYSE410) were cultured in RPMI 1640 (Gibco) with 10% FBS, 50 U mL^−1^ penicillin and 50 U mL^−1^ streptomycin. ZEC-014-1 cell line was cultured in DMEM/F12 (1:1) containing 10% FBS, 1% non-essential amino acid, 50 U mL^−1^ penicillin and 50 U mL^−1^ streptomycin. ESCC cell lines (KYSE180 and KYSE410) were established and provided by Dr. Shimada Yutaka (Faculty of Medicine, Kyoto University)^26^. KYSE150 cell line was purchased from Cell Bank of Type Culture Collection of Chinese Academy of Sciences. ZEC-014-1 cell line was a generous gift from Dr. Dan Su (Zhejiang Cancer Hospital). All cells were tested for mycoplasma contamination and maintained under the humidified 5% CO_2_ atmosphere at 37 °C.

### Knockdown of BRCA1 and BRCA2 in ESCC cell lines

Lentivirus HBLV-U6 carrying four shRNA separately (BRCA1-shRNA1: GAGTATGCAAACAGCTATAAT; BRCA1-shRNA2: TTGCAACCTGAGGTCTATAAA; BRCA2-shRNA1: GCAGCCATTAAATTGTCCATA; BRCA2-shRNA3: CCTCTGAAAGTGGACTGGAAA) targeting BRCA1/2 and the corresponding negative control (NC) siRNA were used to knock down BRCA1/2 in ESCC cell lines (KYSE410 and ZEC-014-1). Briefly, cells with 40–50% confluence were infected with lentivirus supernatant (Hanbio, China). After 48 h incubated at 37 °C, cisplatin (Sigma, P4394) solutions with serial concentrations (2, 5, 10, 30) were added to cells in triplicate. After incubating for another 48 h, cells were subjected to MTT assay. The knockdown efficiency of shRNAs that target to *BRCA1* or *BRCA2* was determined via RT-qPCR by using the primers against *BRCA1* (forward, 5′-GCTACAGAAACCGTGCCAAA-3′; reverse, 5′-TATCCGCTGCTTTGTCCTCA-3′) and *BRCA2* (forward, 5′-TGGAAACTGGCAGCTATGGA-3′; reverse, 5′-TTTTGCAGCTGTGTCATCC-3′).

### Construction of ERBB4 mutants

Twenty-seven mutations including five novel point mutations from 39-Mseq cohort and 22 mutations from the TCGA, early Chinese, and Japanese cohorts distribute at extracellular and kinase domain regions of ErbB4. To investigate their functional importance, we compared their predicted consequence based on evolutionary conservation and nature of the amino acid change. These analyses showed a skewing of functional importance (FI) scores for three mutations (ErbB4-E57K, ErbB4-P377R, and ErbB4-V391I) that were identified from 39-Mseq cohort, supporting their biological relevance and are unlikely to simply be passenger mutations. We then selected these three mutations that located at the kinase domain and had higher FI score and constructed the corresponding mutants using site-directed mutagenesis kit according to the manufacturer’s instructions^58^.

### MTT assay

5 × 10^3^ cells were seeded in 96-well plates and incubated at 37 °C, 5% CO_2_ overnight. After that, cells were incubated with 20 μL of 5 mg mL^−1^ MTT for 4 h in an incubator, then MTT solution was removed and 150 μL DMSO was added to each well. Cells in culture plates were shaken for 10 min at RT to dissolve the MTT crystals, and color intensity was measured by using a Microplate Reader (Bio-Rad) at 490 nm.

### Tissue microarray and immunohistochemistry staining

Tissue microarray blocks were made by using the tissue microarray builder (TC IV, Chloe, Beijing) with 180 tumor/lymph nodes spots and 38 corresponding normal tissue spots (the volume of another 5 tumor tissues and 1 normal tissue were not enough to make TMA). These tissues were formalin-fixed, paraffin-embedded, and cylinders (1.8 mm in diameter) were then punched from defined regions of the tissue block using a tissue microarrayer and then inserted into the microarray blocks. The 4-μm-thick sections were sliced onto gelatin-coated slides with a microtome (Leica, Nussloch, Germany).

Immunohistochemical analysis was performed as previously decribed^59^ by using rabbit anti-PD-L1 monoclonal antibody (ZSGB-BIO, ZA-0629). In brief, sections were incubated with primary antibody at 1:75 dilution for 15 h at 4 °C in wet box, followed by detection with Kit 5020 (Zhongshan, China) and the DAB detection kit (Maixin), forming dark-brown precipitation. Then slides were counterstained with hematoxylin and the images were captured by Aperio Scan Scope at ×40 magnification (Leica, Nussloch, Germany). The immunoreactive *H* score was determined by Aperio ImageScope Membrane v. 9 software. Briefly, the PD-L1 expression was evaluated on the whole spots, TCs, and tumor-infiltrating ICs. For TC or IC, the proportion of PD-L1-positive cells was estimated as the percentage of total TCs or tumor-infiltrating ICs. According to the quantification criteria of clone SP142 antibody, the positive staining was defined as the proportion equal to or greater than 1%^60^.

### FISH analysis

Tissue microarray slides of interesting ESCC cases were incubated with probe against PD-L1, which conjugated with FITC or that against centromere 9, which conjugated with TRITC (F. 01256, Linked-Biotech pathology, Guangzhou). Staining was performed as the manufacturer’s instruction. Samples were then observed through an upright microscope (Zeiss Axio-ImagerZ.1) with DAPI, FITC, and Rhodamine filter cubes. Images were captured by the AxioCamMRm CCD camera and Axiovision version 4.5 software.

### Immunoblotting

To assess the activation of *ERBB4* mutations, wild type and mutational *ERBB4* were cloned into pLenti-EF1а-ERBB4-CMV-GFP vector, and the viruses were produced by transfecting of HEK293T cells with the packaging plasmids along with the lentiviral vectors (PolePolar Biotechnology, China) by using Lipofectamine 2000 reagent (Invitrogen), medium was changed after 6 h. After 24 h, viruses were harvested and passed through 0.22 μm filter. Then HEK293T, KYSE150, and KYSE180 cells were infected with lentiviruses^10^. After confirming the expression of GFP, cells were starved overnight and stimulated with 50 ng mL^−1^ hNRG-1 (Cell Signaling Technology, #5218) for 10 min in an incubator, then lysed for 30 min with lysis buffer (1% Triton X-100, 50 mM Tris-HCl, pH 7.6, 150 mM NaCl, 1% sodium deoxycholate and 0.1% sodium dodecyl sulfate (SDS)) supplemented with phosphatase inhibitor (PhosSTOP, Roche). Lysates were cleared by centrifugation at 15,000 × *g*, 4 °C for 20 min, and the protein concentration was determined by the Bradford method^10^. Fifty micrograms protein was loaded for electrophoresis in SDS-polyacrylamide gel electrophoresis and immunoblotting was performed by using anti-ErbB4 (Abcam, ab32375) and anti-pErbB4 (Abcam, ab1092723) antibodies. Either horseradish-peroxidase (HRP)-conjugated anti-rabbit or anti-mouse IgG (Cell Signaling Technology, #7074) was used to detect the primary antibodies. Then the bound antibodies were detected using Western Lighting plus-ECL reagent (PerkinElmer) and recorded by a LAS4000 device (Fuji). The blots with uncropped molecular weight markers are supplied in the Source Data.

### Colony formation assay

Colony formation was performed as previously described^9^. Briefly, cells were seeded in a 60 mm culture plate with 1000 cells and incubated at 37 °C, 5% CO_2_ for 7 days. On day 7, cells were fixed with 4% polyformaldehyde for 15 min and stained with 1% crystal violet. The experiment was done and quantified in triplicate.

### Statistical analysis

Statistical analysis was performed using R software (version R 3.2.3). The two-sided Student’s *t*-test was used to compare the significant differences between two groups. The Fisher’s exact test was used to determine the association between two categories. Statistical significance was declared if the *P*-value was <0.05.

## Supplementary information

Supplementary Information

Description of Additional Supplementary Files

Supplementary Data 1

Supplementary Data 2

Supplementary Data 3

Supplementary Data 4

Supplementary Data 5

Supplementary Data 6

Supplementary Data 7

Source data

## Data Availability

All data files supporting the conclusions of this project are going to be uploaded to Sequence Read Archive (SRA) under accession code PRJNA511368. The source data underlying Figs. 1e, 4d and Supplementary Figs. 4d, 9b, c are provided as a Source Data file. All other data are available from the corresponding authors upon reasonable request.

## Code availability

All the related algorithms were described above and additional code used in this study is available upon request.
