# Author Correction: Multi-region sequencing unveils novel actionable targets and spatial heterogeneity in esophageal squamous cell carcinoma

**DOI:** 10.1038/s41467-020-19641-9

**Published:** 2020-11-12

**Authors:** Ting Yan, Heyang Cui, Yong Zhou, Bin Yang, Pengzhou Kong, Yingchun Zhang, Yiqian Liu, Bin Wang, Yikun Cheng, Jiayi Li, Shixing Guo, Enwei Xu, Huijuan Liu, Caixia Cheng, Ling Zhang, Ling Chen, Xiaofei Zhuang, Yu Qian, Jian Yang, Yanchun Ma, Hongyi Li, Fang Wang, Jing Liu, Xuefeng Liu, Dan Su, Yan Wang, Ruifang Sun, Shiping Guo, Yaoping Li, Xiaolong Cheng, Zhihua Liu, Qimin Zhan, Yongping Cui

**Affiliations:** 1 Shenzhen Peking University-The Hong Kong University of Science and Technology (PKU-HKUST) Medical Center, Peking University Shenzhen Hospital, 518035 Shenzhen, PR China; 2 Department of Pathology & Shanxi Key Laboratory of Carcinogenesis and Translational Research on Esophageal Cancer, Shanxi Medical University, 030001 Taiyuan, PR China; 3 Department of Tumor Surgery, Shanxi Cancer Hospital, 030013 Taiyuan, PR China; 4 College of Information and Computer, Taiyuan University of Technology, 030001 Taiyuan, PR China; 5 College of Letter & Science, University of California Berkeley, Berkeley, CA 94704 USA; 6 Anglo-Chinese School (Independent), Singapore, 139650 Singapore; 7 Department of Pathology, Shanxi Cancer Hospital, 030013 Taiyuan, PR China; 8 Department of Pathology, the First Hospital, Shanxi Medical University, 030001 Taiyuan, PR China; 9 Department of Pathology, Tianjin Central Hospital of Gynecology Obstetrics, Nankai University Gynecology Obstetrics Hospital, 300052 Tianjin, PR China; 10 Department of General Surgery, the First Hospital, Shanxi Medical University, 030001 Taiyuan, PR China; 11 Department of Pathology, Zhejiang Cancer Hospital, 310022 Hangzhou, PR China; 12 Key laboratory of Carcinogenesis and Translational Research (Ministry of Education/Beijing), Laboratory of Molecular Oncology, Peking University Cancer Hospital & Institute, 100191 Beijing, PR China; 13 Tumor Biobank, Shanxi Cancer Hospital, 030013 Taiyuan, PR China; 14 Department of Colorectal & Anal Surgery, Affiliated Provincial Hospital of Shanxi Medical University, 030001 Taiyuan, PR China; 15 State Key Laboratory of Molecular Oncology, National Cancer Center/Cancer Hospital, Chinese Academy of Medical Sciences and Peking Union Medical College, 100021 Beijing, PR China

**Keywords:** Cancer genomics, Tumour heterogeneity

Correction to: *Nature Communications* 10.1038/s41467-019-09255-1, published online 11 April 2019.

This Article contained errors in Fig. 1 and Supplementary Fig. 4. In Fig. 1. and Supplementary Fig. 4d the gels were inappropriately spliced together. For each experiment, multiple gels were prepared and the gels were probed in parallel. The figure legends have been updated to describe the parallel processing of the blots.

Additionally, in Supplementary Fig. 4d the colony formation image for ErbB4-E57K of the KYSE180 cells was inadvertently duplicated from the ErbB4-WT image. The image has now been replaced, while the experiment was repeated three times, images are only available from one experiment.

Finally, the original manuscript was published without the Source data for the histograms and gels. The Source data file now contains all the Source data supporting the study.

These errors have been corrected in the Pdf and HTML versions of the Article.

