# Supplementary material for: Multi-region sequencing unveils novel actionable targets and spatial heterogeneity in esophageal squamous cell carcinoma
Source: Nat Commun. 2019 Apr 11;10:1670. doi: 10.1038/s41467-019-09255-1 (PMC6459928; doi:10.1038/s41467-019-09255-1)
Supplement: Supplementary file 1 — Supplementary Information [file 41467_2019_9255_MOESM1_ESM.pdf]

Supplementary information

Multi-region sequencing unveils novel actionable targets and spatial heterogeneity in esophageal squamous cell carcinoma

Yan et al.

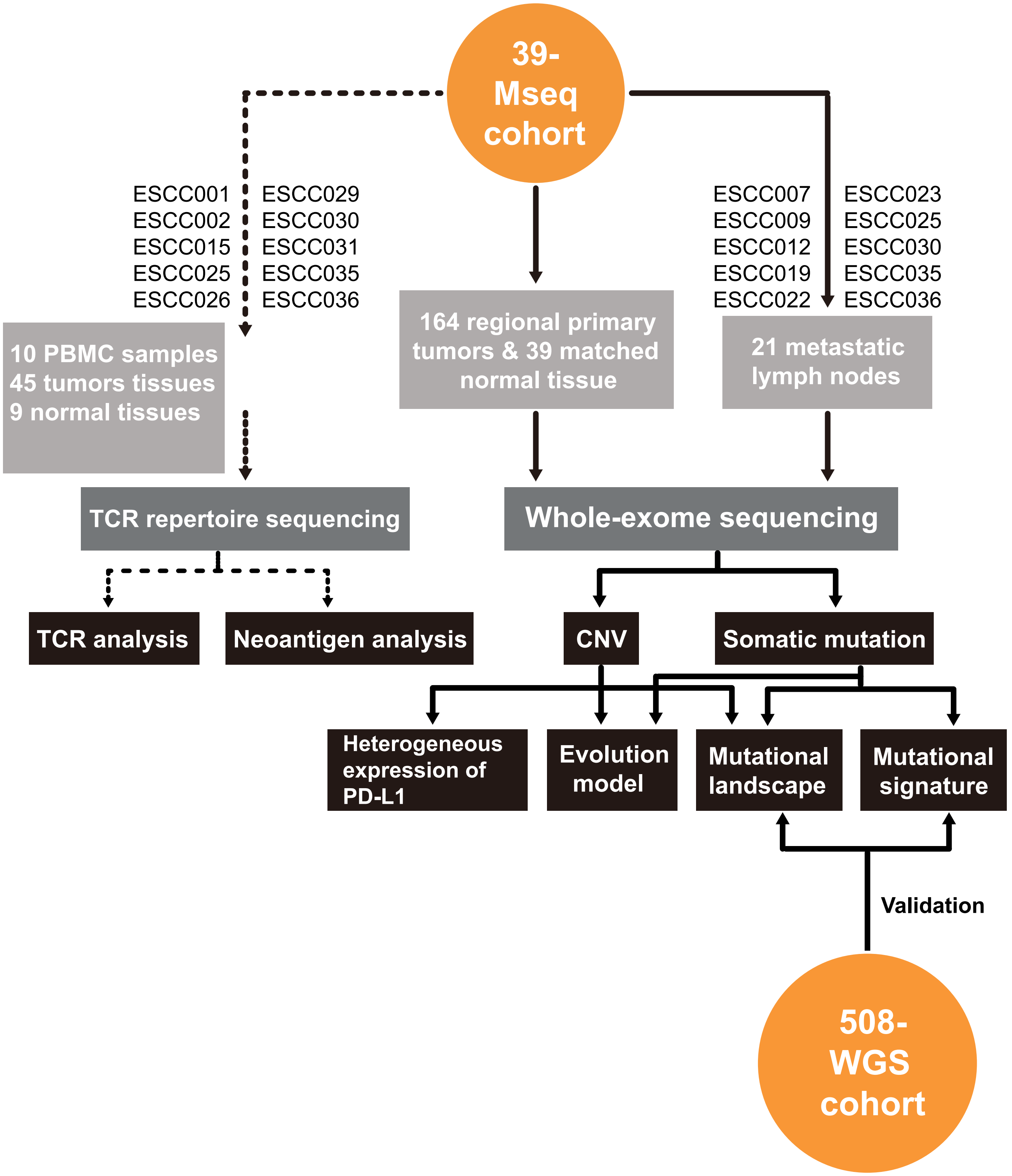

Supplementary Figure 1. A flow chart shows the relationship between different cohort and studies.

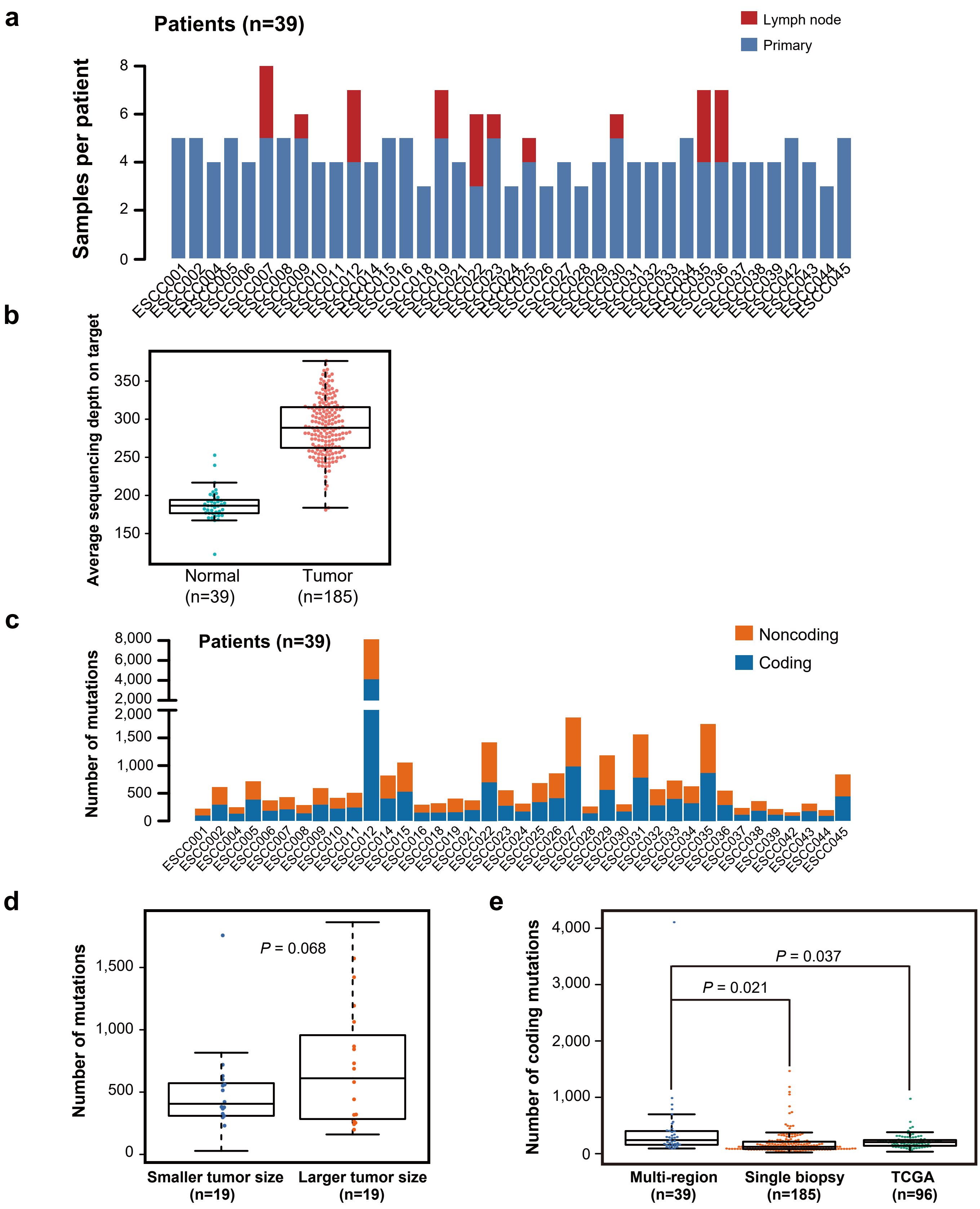

Supplementary Figure 2. Landscape of mutations of 39 ESCC cohort. (a) Number of specimens within 39 patients. (b) Sequencing depth of normal and tumor samples. (c) Number of coding and non-coding mutations across 39 ESCCs. (d) Comparison of mutation numbers in ESCC with different tumor size. (e) Comparison of mutation numbers between multi-sequencing, single biopsy and TCGA cohort.

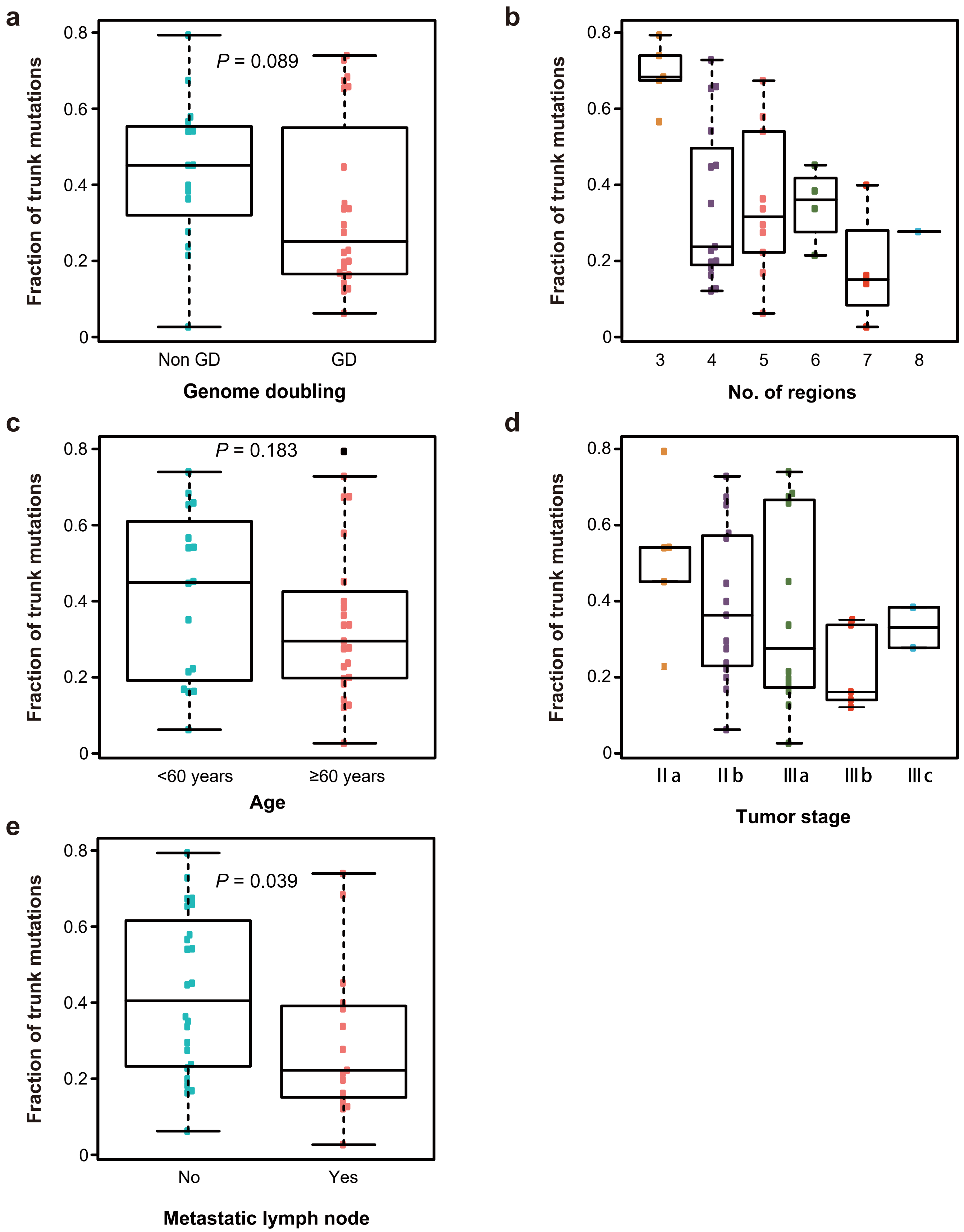

Supplementary Figure 3. Mutation intra-tumor heterogeneity by clinical information. Fraction of trunk mutations by genome doubling **(a)**, number of regions **(b)**, age **(c)**, tumor stage **(d)** and metastatic lymph node **(e)**.

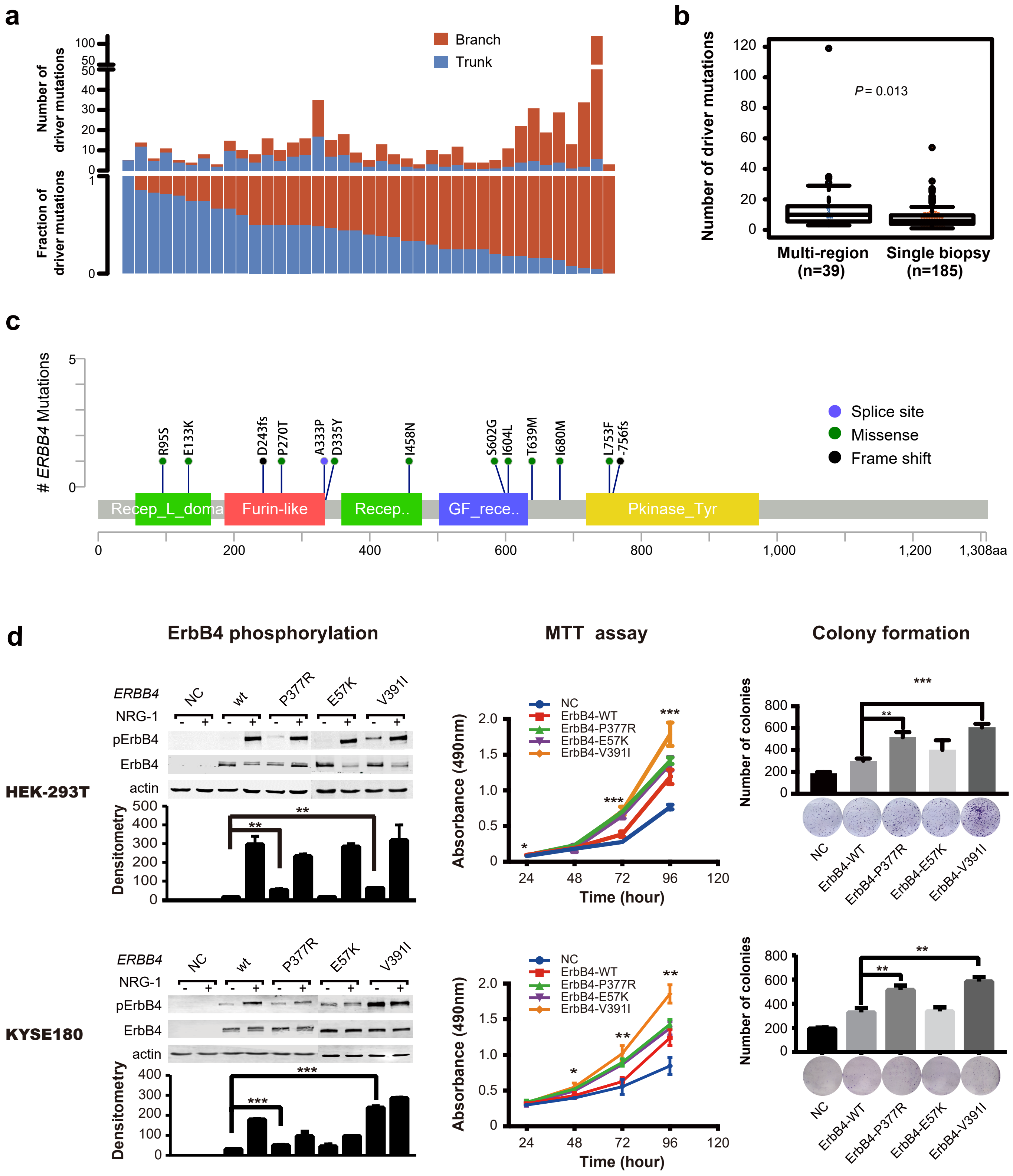

Supplementary Figure 4. Clonal status of driver mutations in ESCC. (a) Top panel shows number of drivermutations detected in each patient, and bottom panel shows the percentages of trunk or branch driver mutations. (b) Comparison of driver mutation numbers between multi sequencing and single biopsy. (c) Schematics of ErbB4 protein alterations resulted from mutations identified in 508-WGS ESCC cohorts. (d) The effect of ERBB4 wild-type and mutants on ErbB4 phosphorylation and cell proliferation as monitored by NRG-1 stimulation (left), MTT (middle) and colony formation assay (right), respectively. Experiments were performed with HEK293T cells (upper), and KYSE180 cells (lower) in triplicate and all data are mean ± SD. Student *t*-test. $*P < 0.05$, $**P < 0.01$, $***P < 0.001$. Source data are provided as a Source Data file.

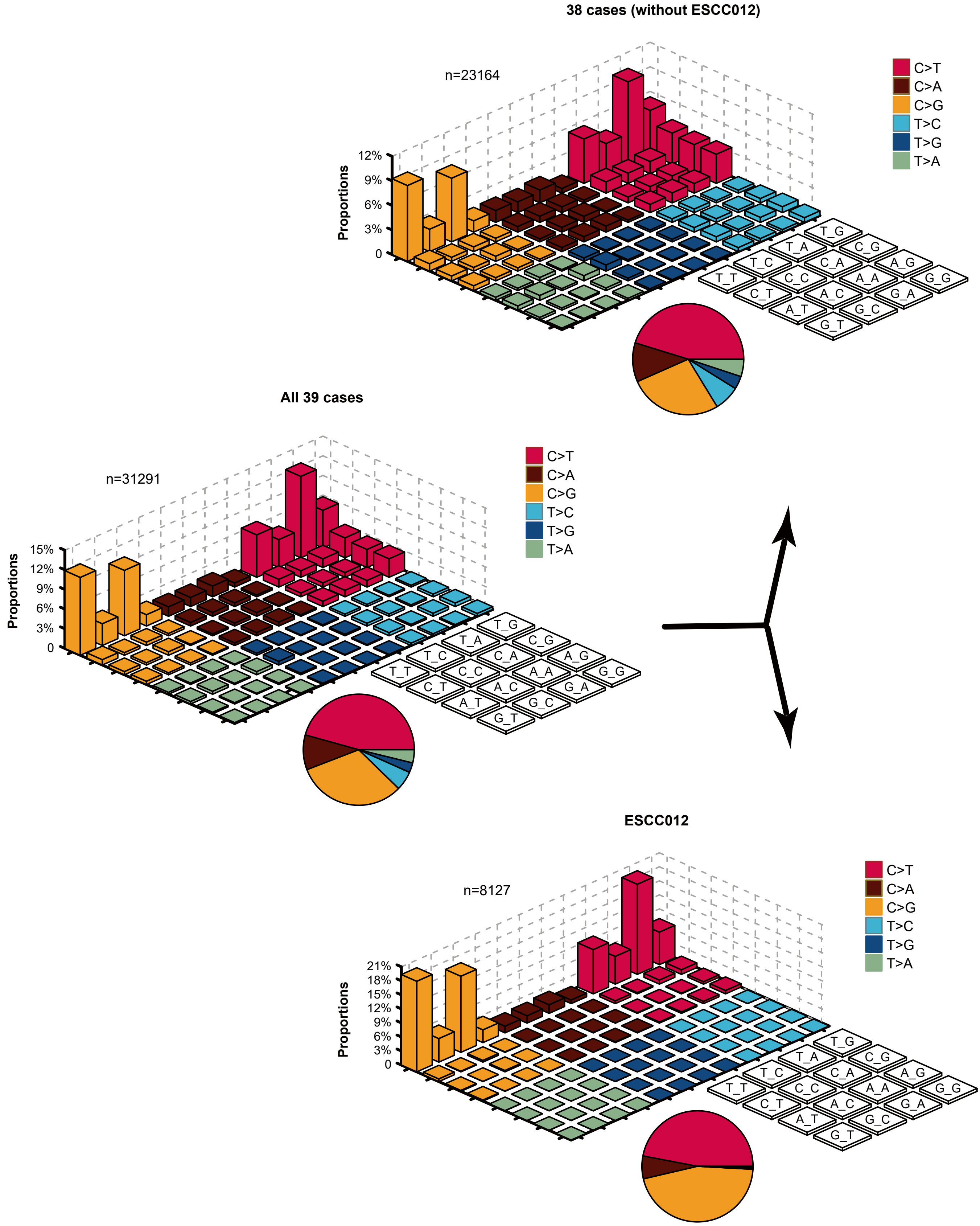

Supplementary Figure 5. Mutation-spectrum analysis across 39 ESCCs (ESCC012 and other 38 ESCCs). Base substitutions were divided into six subtypes to represent the six possible base changes (each subtype is represented by a different color). The proportion was calculated as the number of somatic SNVs divided by the number of somatic SNVs that contained the specified trinucleotide.

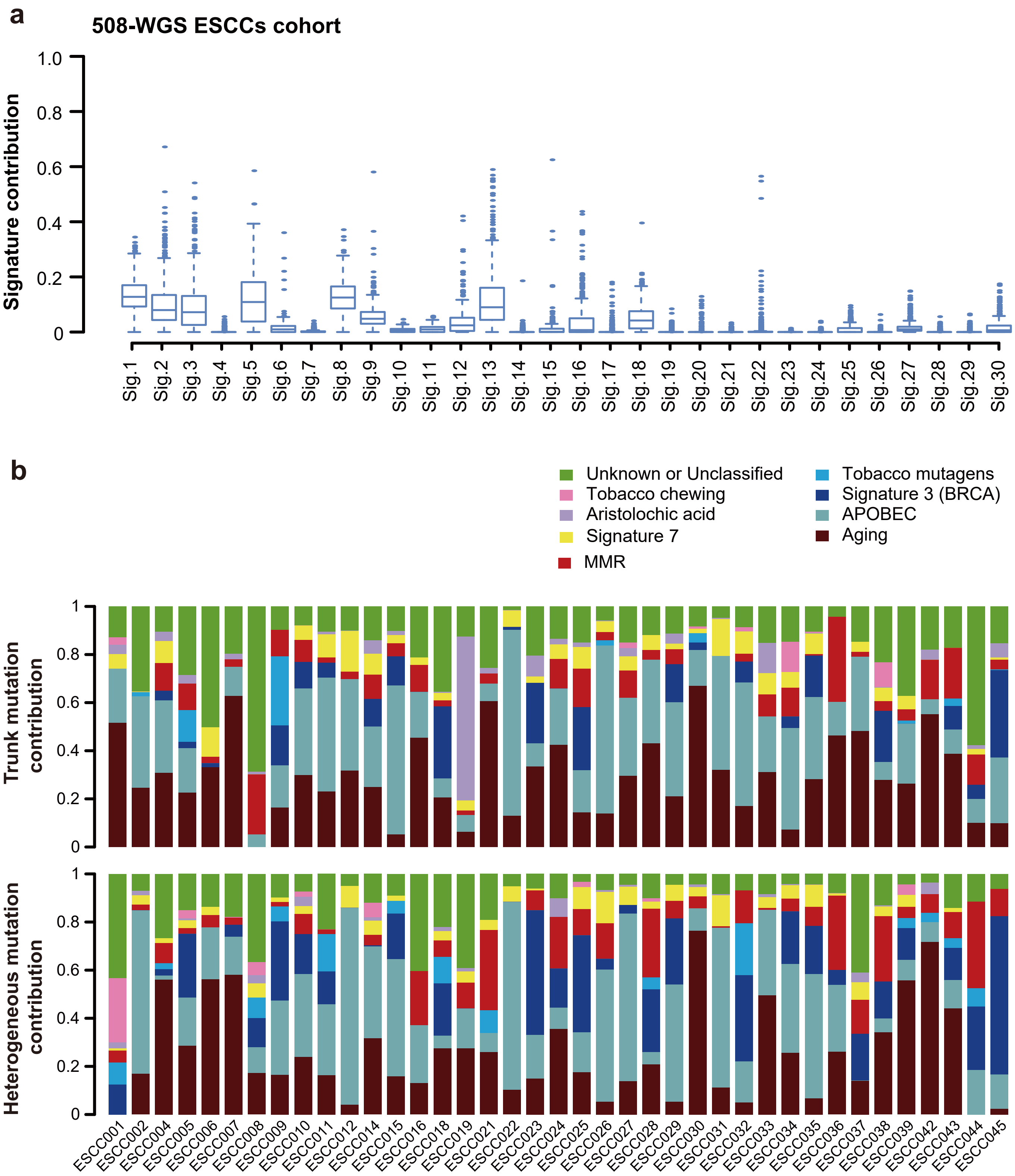

Supplementary Figure 6. Summary of mutational signatures in 508 and 39 ESCCs cohort. (a) The relative contribution of each mutational signature across 508 ESCCs. **(b)** Mutational signatures identified across 39 individuals, split to trunk and heterogeneous according to timing of mutations.

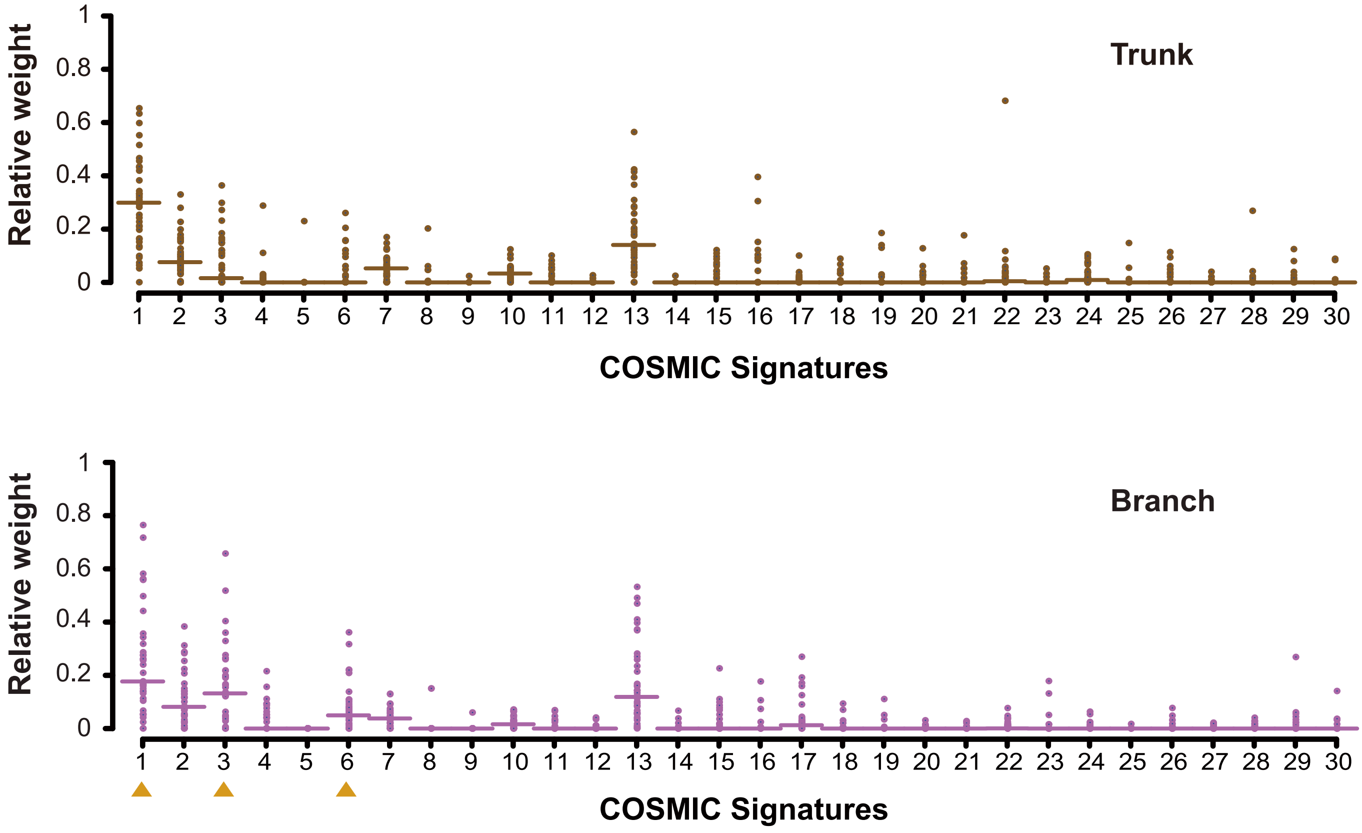

Supplementary Figure 7. Relative weight of 30 COSMIC signatures across 39 ESCCs identified from trunk (upper) or heterogeneous (lower) mutations.

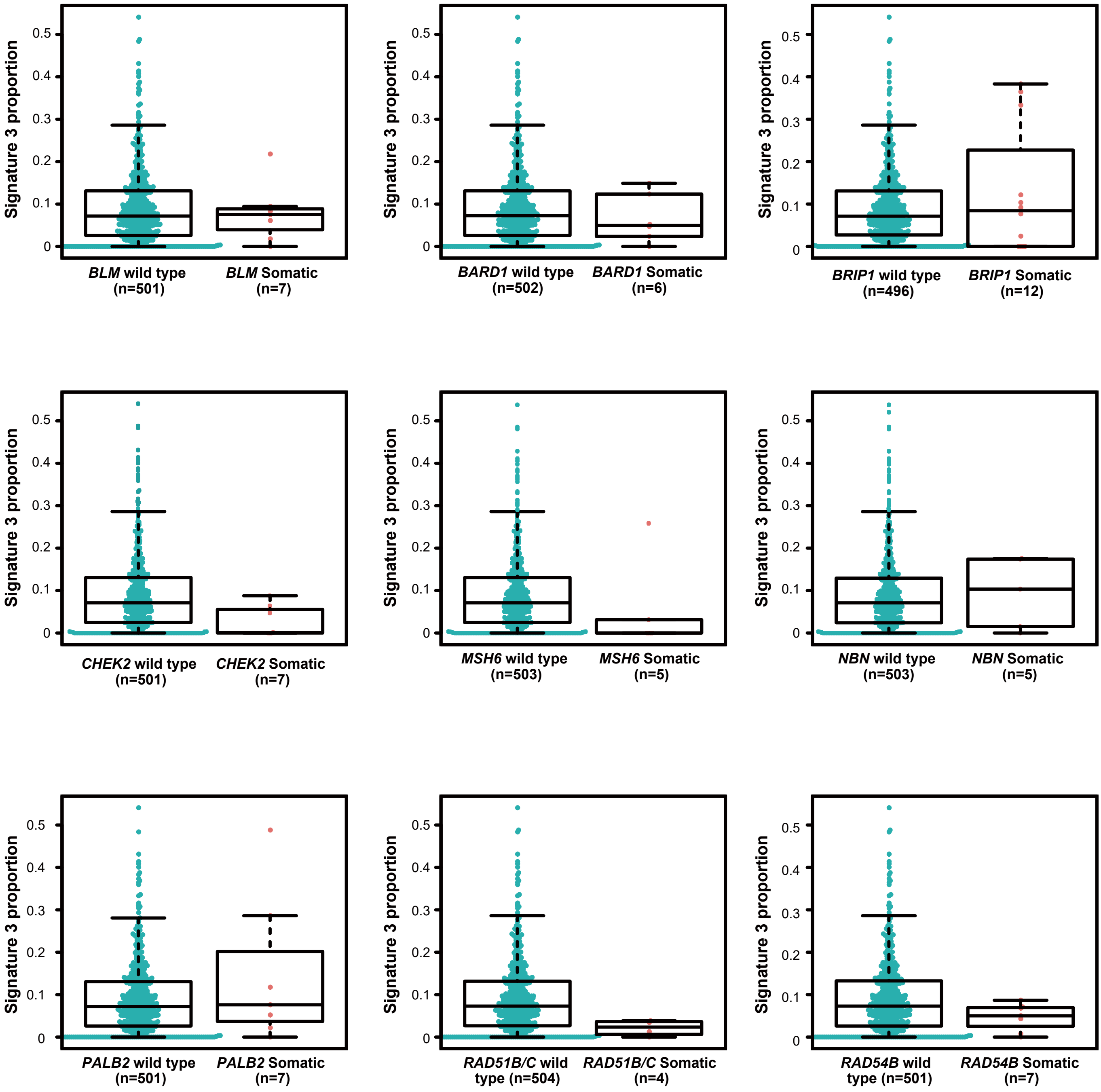

Supplementary Figure 8. Signature 3 activity association with mutation of HR components beyond *BRCA1/2.*

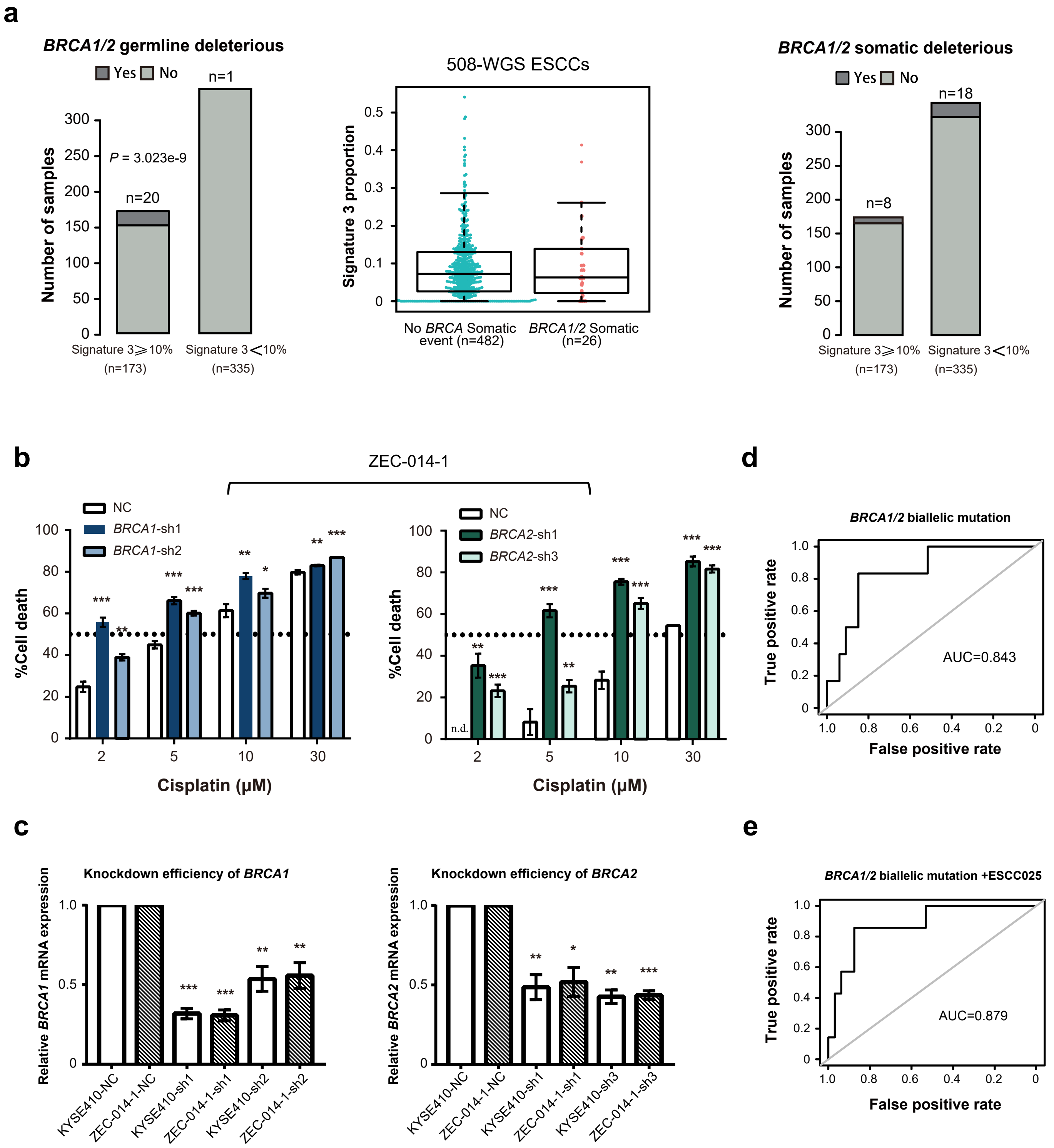

Supplementary Figure 9. *BRCA1/2* mutation is related to cisplatin sensitivity in ESCC. (a) Middle panel depicts comparison of signature 3 activity in 508-WGS ESCCs with or without *BRCA1/2* somatic mutation (middle). Bar plots show numbers of *BRCA1/2* germline mutation (left) or somatic mutation (right) in 508-WGS ESCCs with high signature 3 activity (relative contribution >10%) and low signature 3 activity (relative contribution<10%). Fisher test. **(b)** Knockdown of *BRCA1* or *BRCA2* significantly increased the sensitivity of ZEC-014-1 cells to cisplatin. Student *t*-test. ∗*P*<0.05, ∗∗*P*<0.01, ∗∗∗*P*<0.001, n.d. means not detected. **(c)** Knockdown effeciency of shRNA targeted to *BRCA1* or *BRCA2* in KYSE410 and ZEC-014-1 cell lines, respectively. Student *t*-test. Error bars are defined as s.d. Source data are provided as a Source Data file. **(d-e)** Receiver operating characteristic (ROC) curves for *BRCA1/2* biallelic mutation predicted on the basis of signature 3 levels in our cohort.

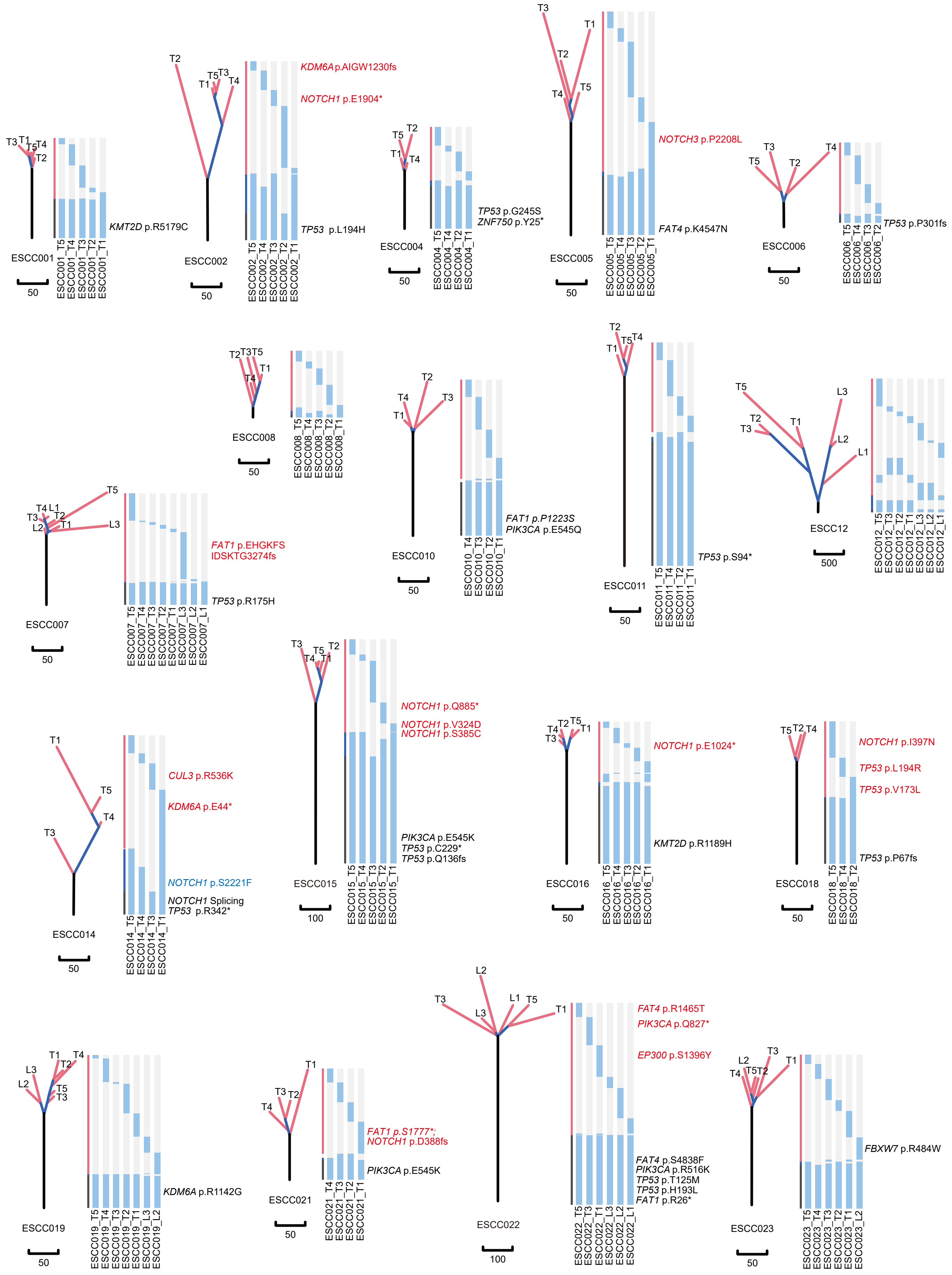

Supplementary Figure 10. Cancer evolutionary tree based on multi-regional sequencing. Left panel displays the phylogenetic tree for each case: trunk, branch, private mutations are colored in dark, blue and red respectively; right panel represents the distribution of the nonsilent-mutations in each region.

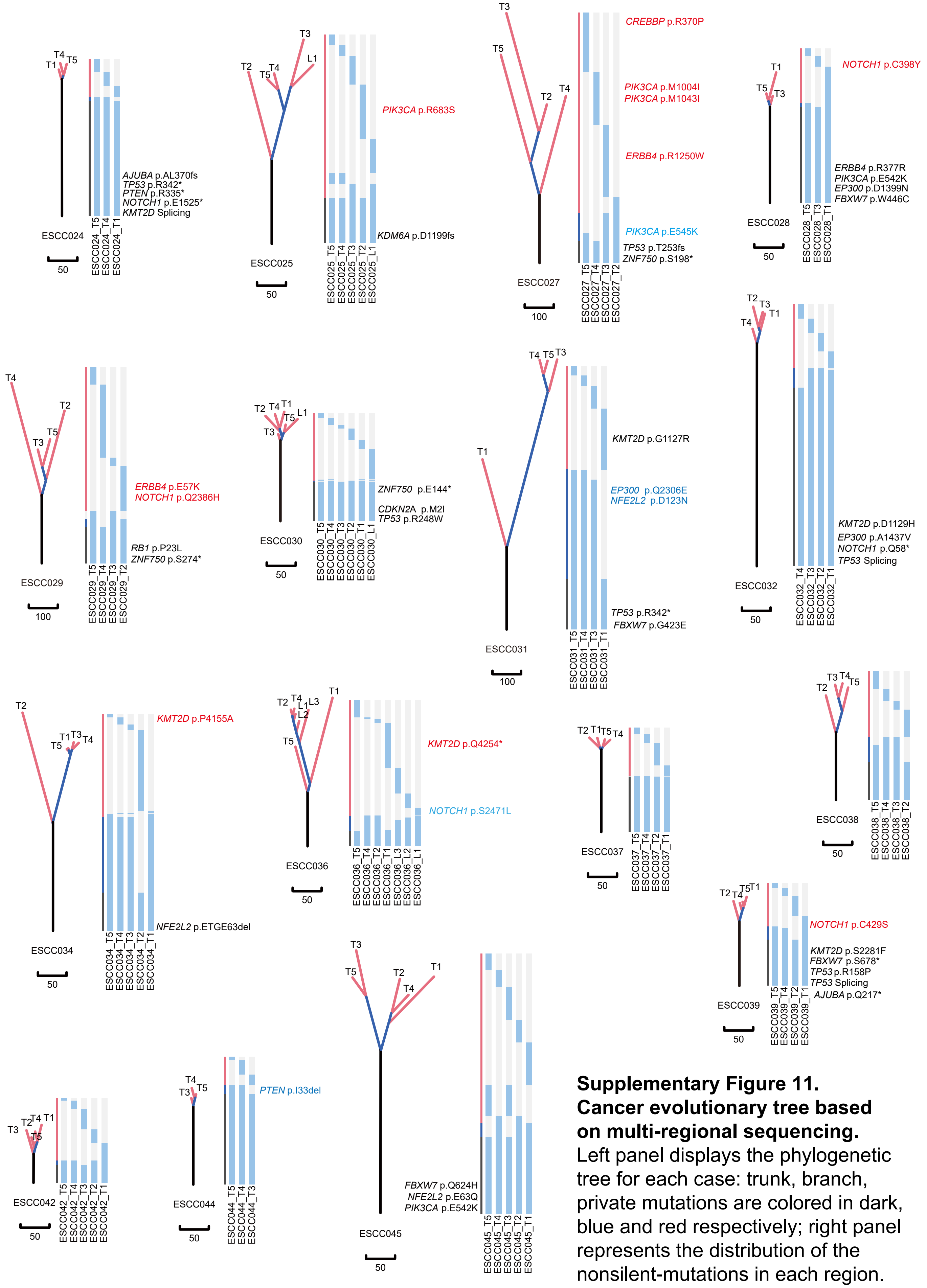

Supplementary Figure 11. Cancer evolutionary tree based on multi-regional sequencing. Left panel displays the phylogenetic tree for each case: trunk, branch, private mutations are colored in dark, blue and red respectively; right panel represents the distribution of the nonsilent-mutations in each region.

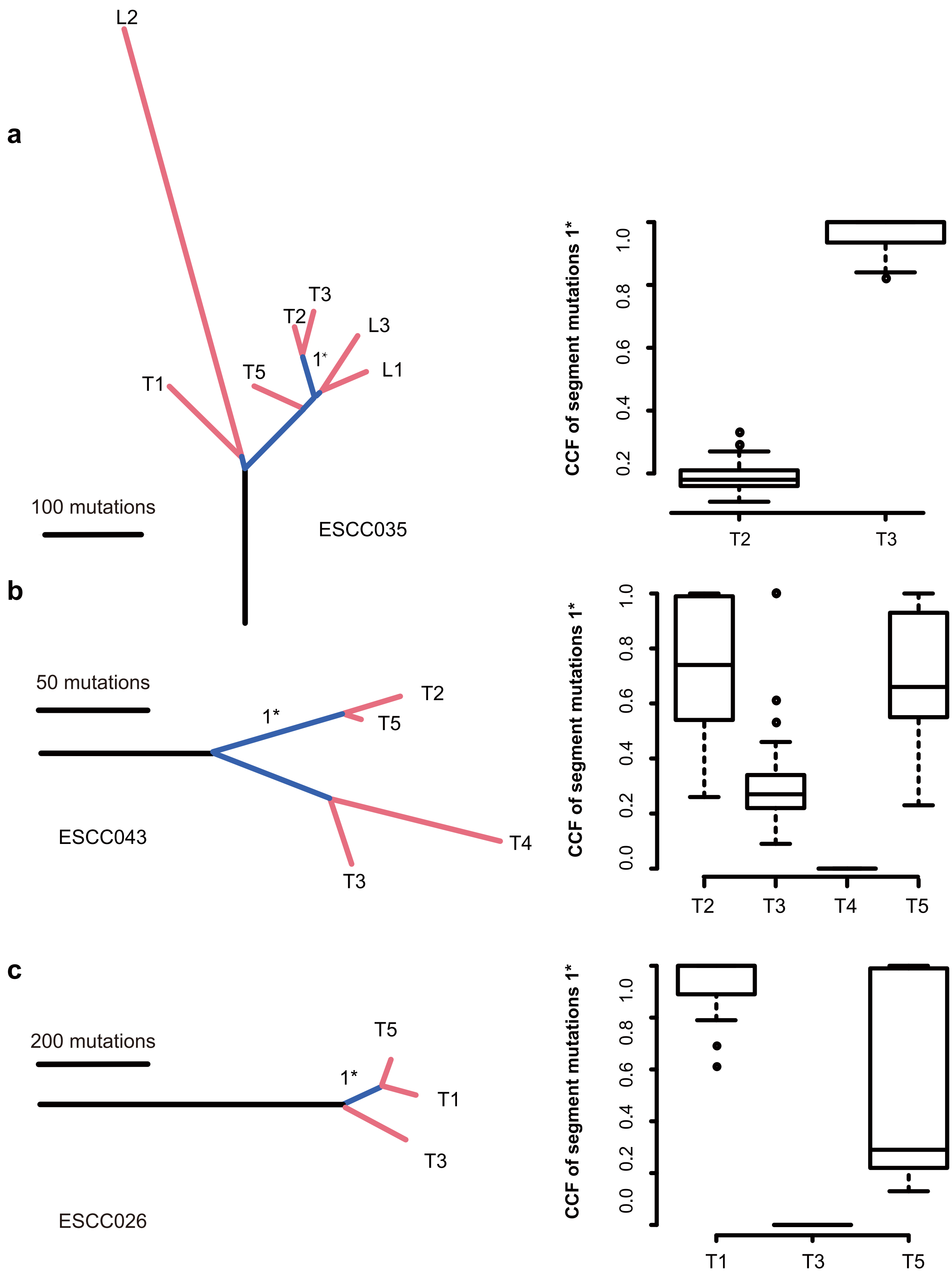

Supplementary Figure 12. Clonal evolution in case ESCC035 **(a)**, ESCC043 **(b)** and ESCC026 **(c)**. Left panel displays the phylogenetic tree; right panel shows the cancer cell fraction of intermixed mutations, respectively.

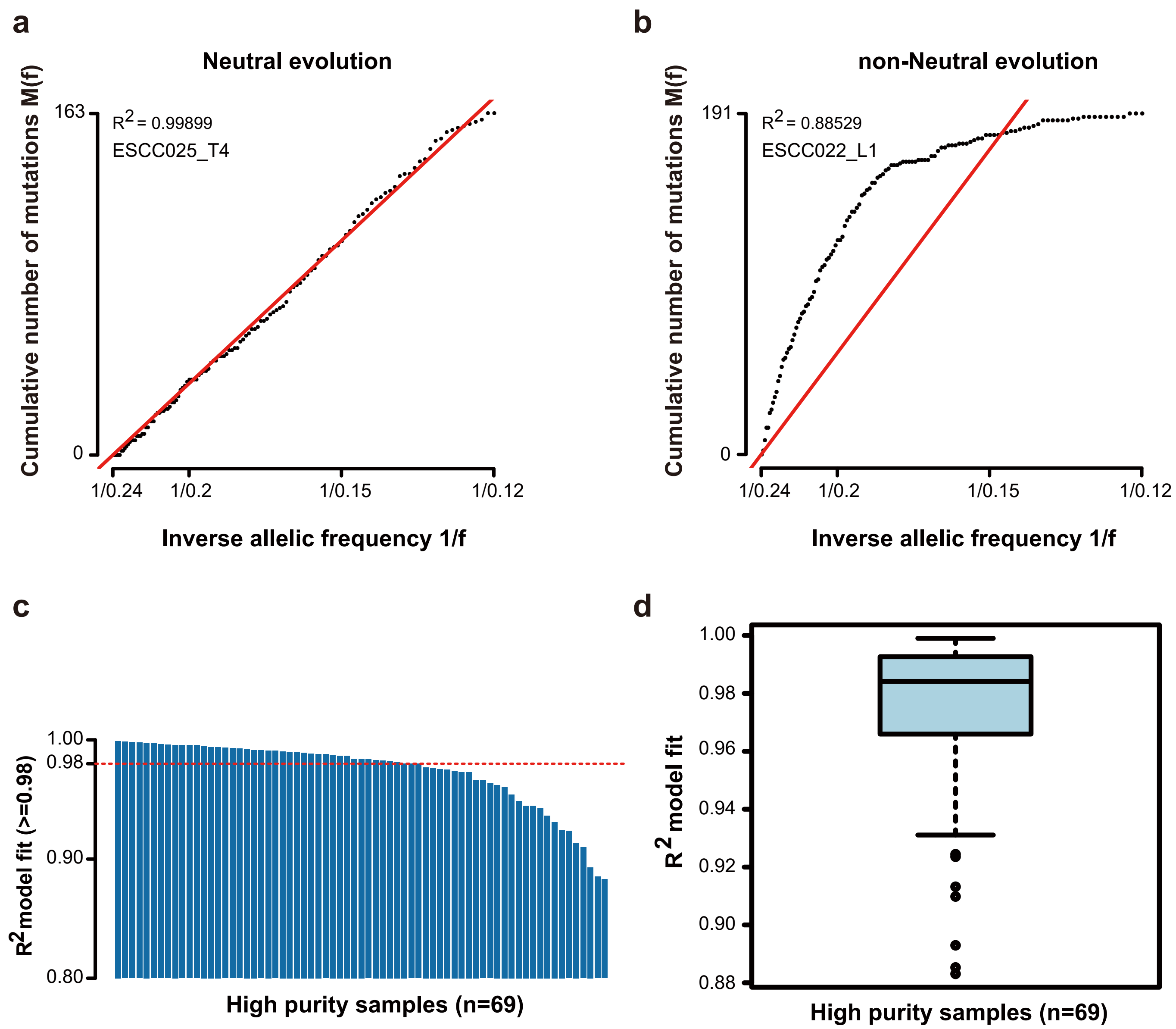

Supplementary Figure 13. Neutral evolution in ESCC. (a) The cumulative distribution of subclonal mutations in ESCC025_T4 was found to be linear with 1/f, precisely as predicted by the neutral model. **(b)** Non-neutral evolution in ESCC022_L1. **(c-d)** The R^2 goodness-of-fit measure for our ESCC cohort. The red line indecates the R^2 = 0.98 threshold for distinguishing neutral from non-neutral tumors.

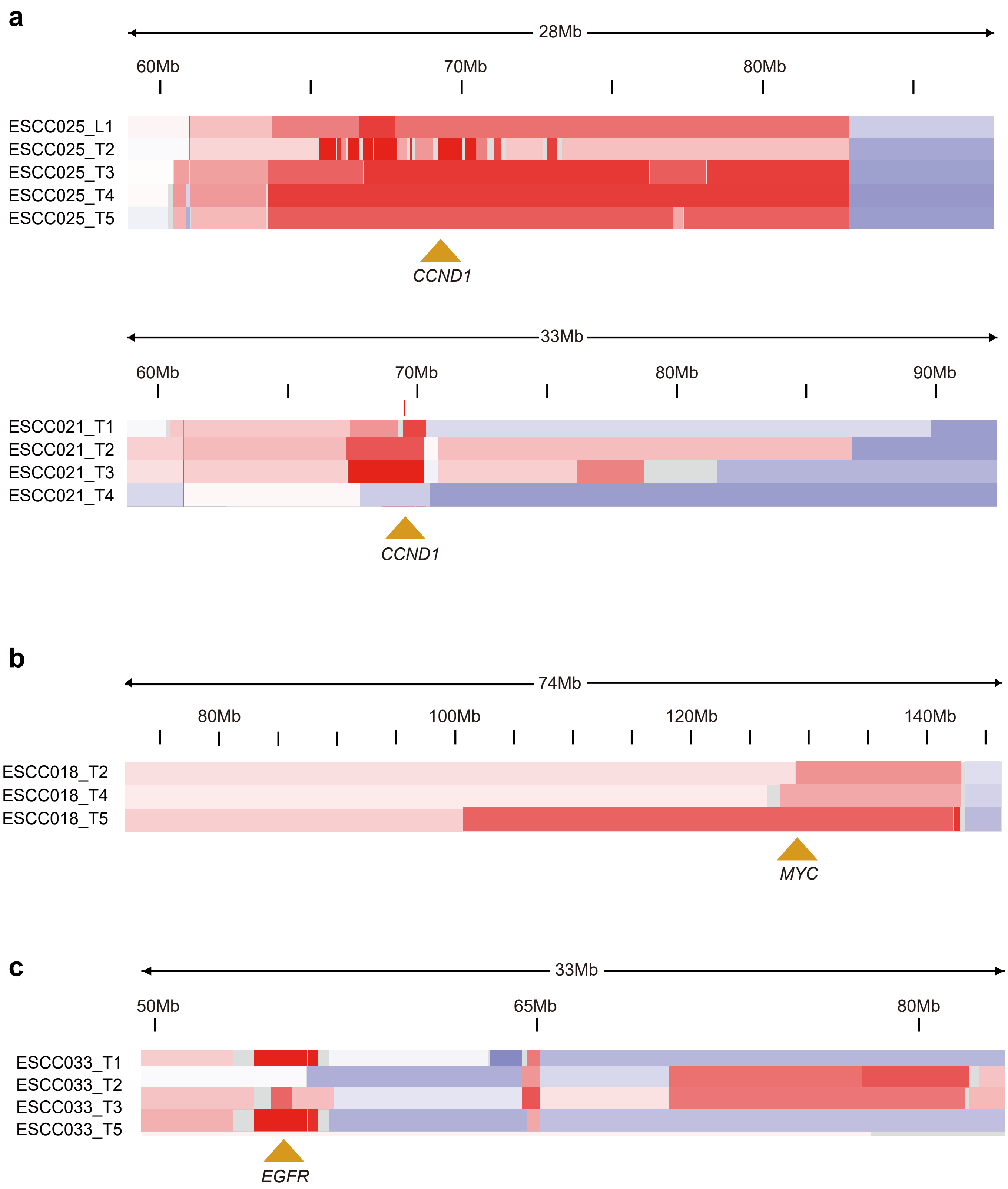

Supplementary Figure 14. Parallel evolution of driver amplified genes. Amplifications and deletions are colored in red and blue, respectively. **(a)** Parallel evolution of focal *CCND1* amplifications in case ESCC025 and ESCC021. **(b)** Parallel evolution of *MYC* amplifications in case ESCC018, of which focal amplification occurred in T2 and T4, whereas T5 had a broad amplification. **(c)** Parallel evolution of *EGFR* amplifications in case ESCC033.

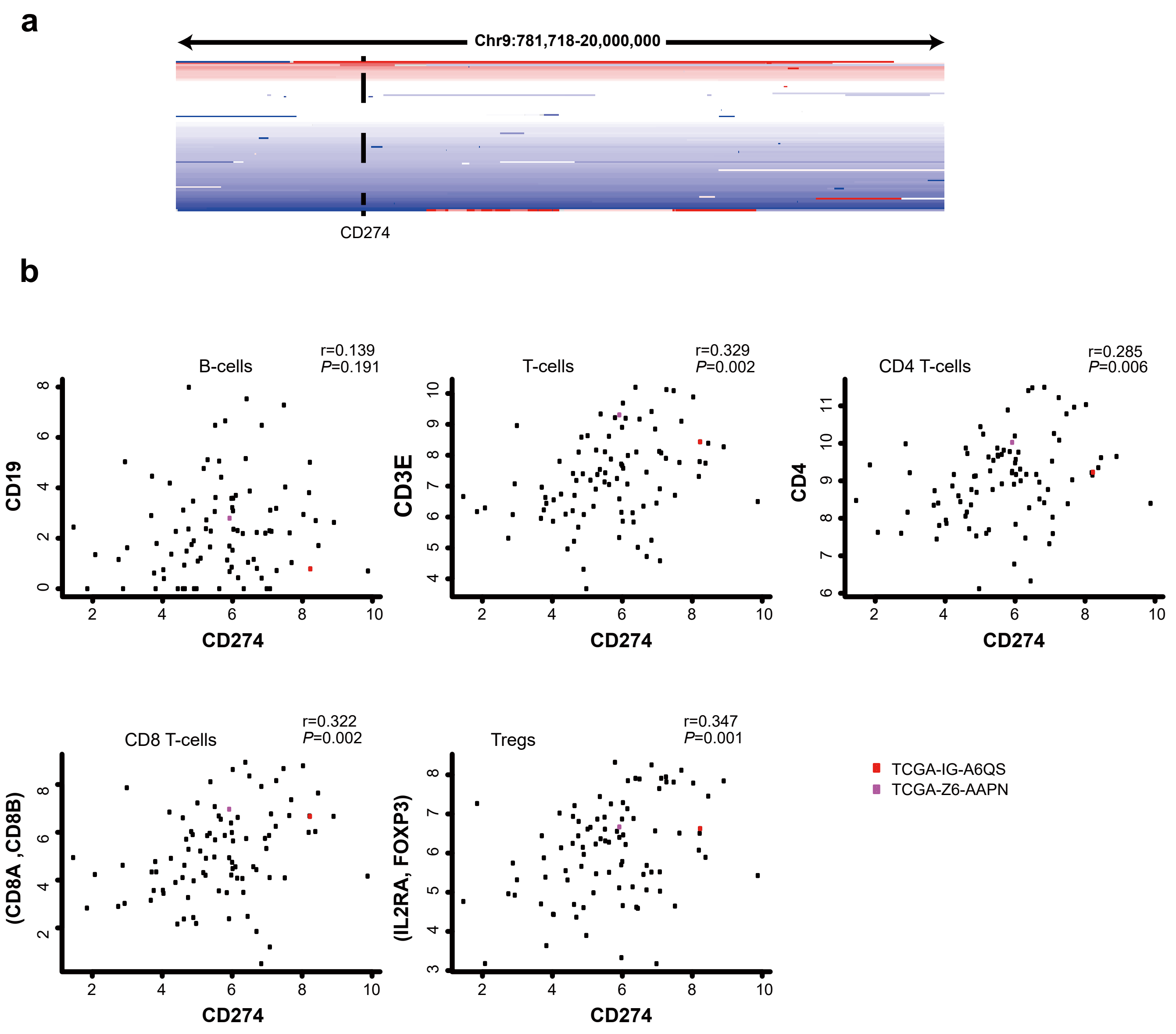

Supplementary Figure 15. Amplification and expression of CD274 in TCGA ESCC data. (a) Copy number changes of CD274 in 90 ESCCs data. **(b)** Transcript level of immune cell related markers in two focal amplified cases.

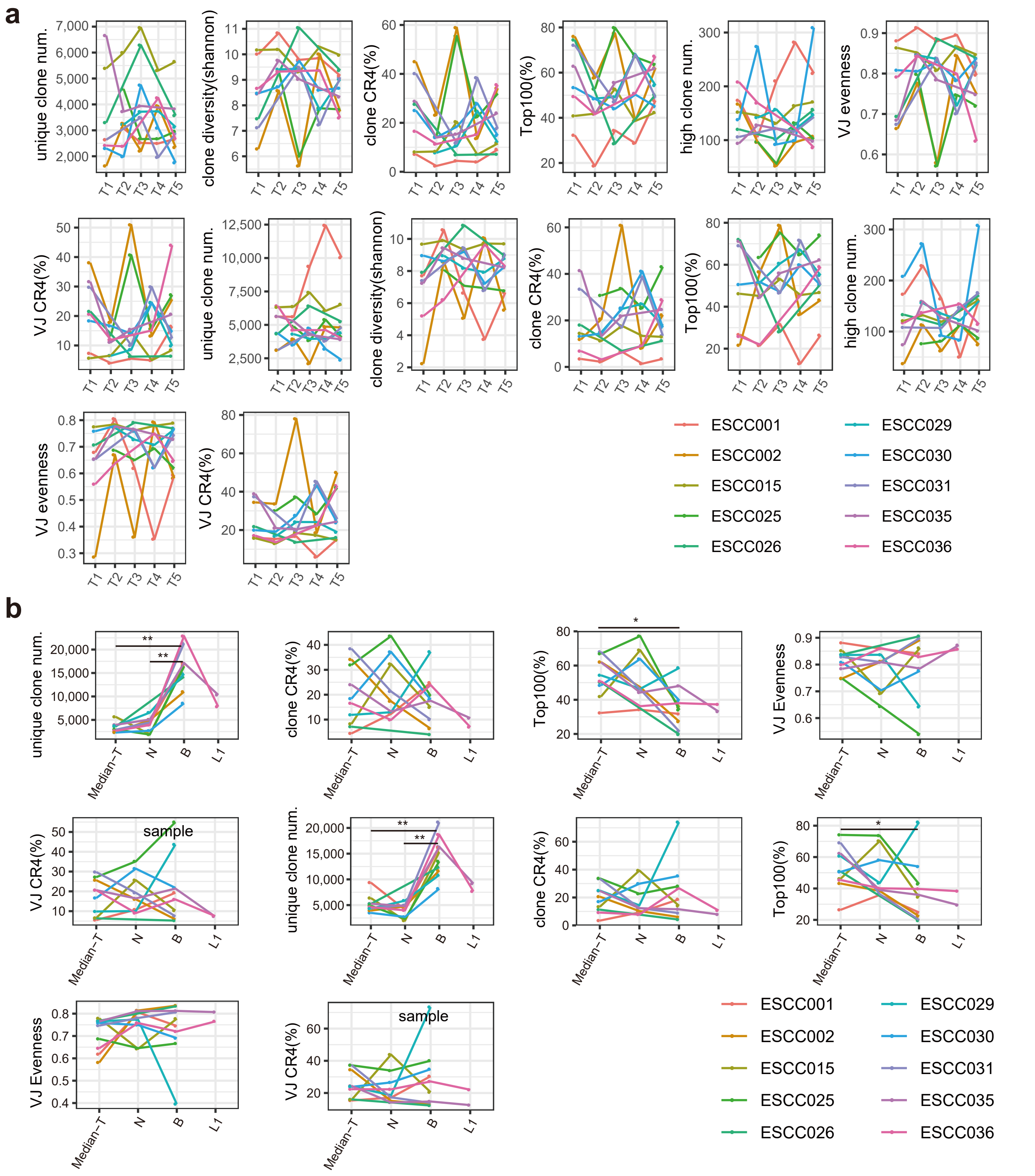

Supplementary Figure 16. Overall diversity among multiple samples. (a) Overall diversity and high frequent clones among multiple tumor tissues. The top panel is the results of TRA and the bottom panel is the results of TRB. **(b)** Overall diversity and high frequent clones among different type of samples (top panel: TRA, bottom panel: TRB). To normalize samples, all statistics here are from the same size effective sequences. unique clone num.: the number of unique clones. Clone diversity: calculated by Shannon index. Clone CR4: the sum of top 4 clones' frequencies. Top100: accumulate frequency of top 10 clones. High clone num.: the number of high frequent (>0.1%) clones. VJ evenness: calculated by Peilou. VJ CR4: the sum of top 4 V-J gene pairings' frequencies. Median-T: the median value of multiple tumor tissues. N, adjacent normal tissue. B, blood. L1, metastatic tissue.

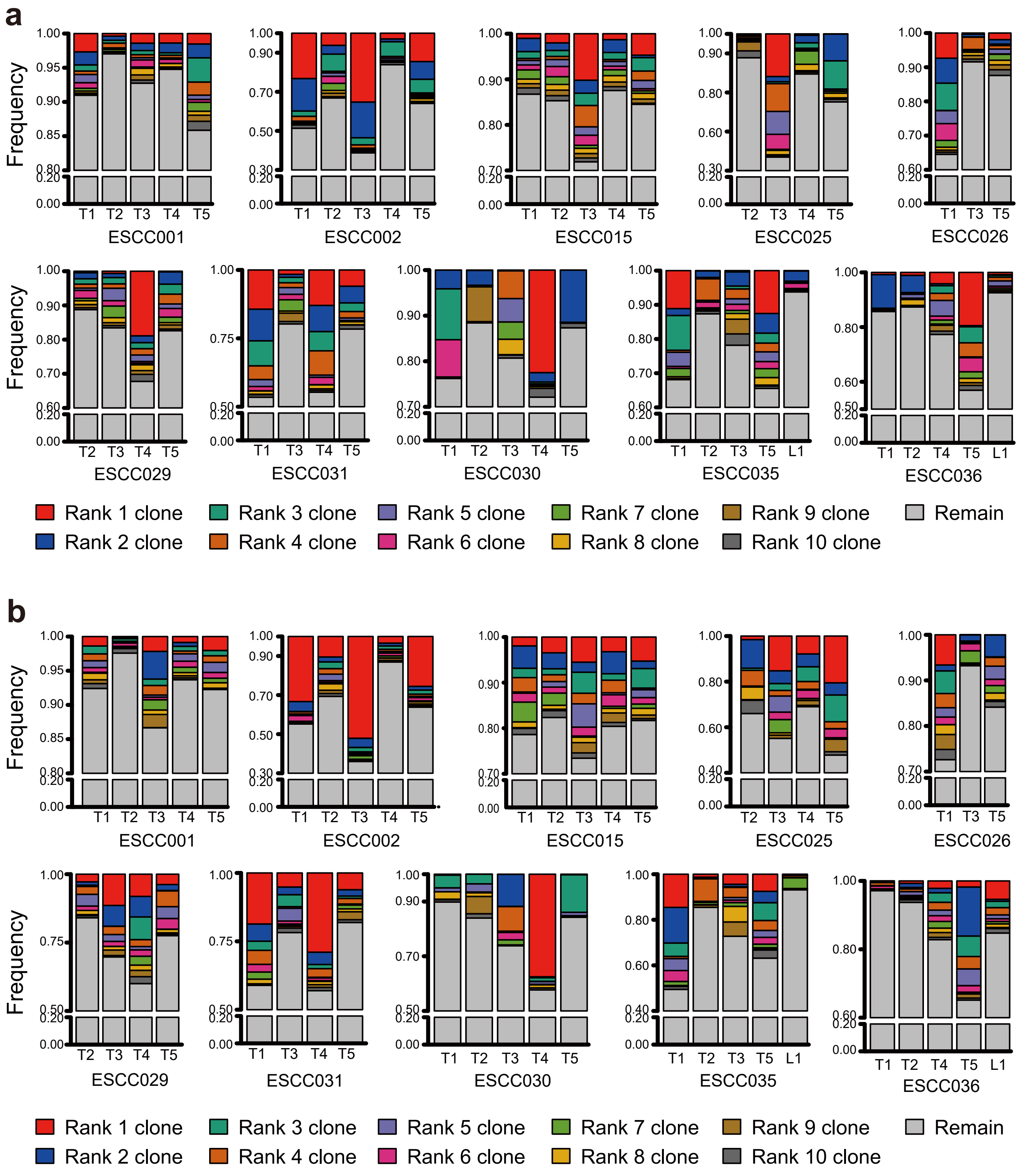

Supplementary Figure 17. The top 10 clones within all regions of tumor. (a) The results of TRA repertoire. **(b)** The results of TRB repertoire. The top 10 most frequent clones in each patient are colored. Grey represents the other clones.

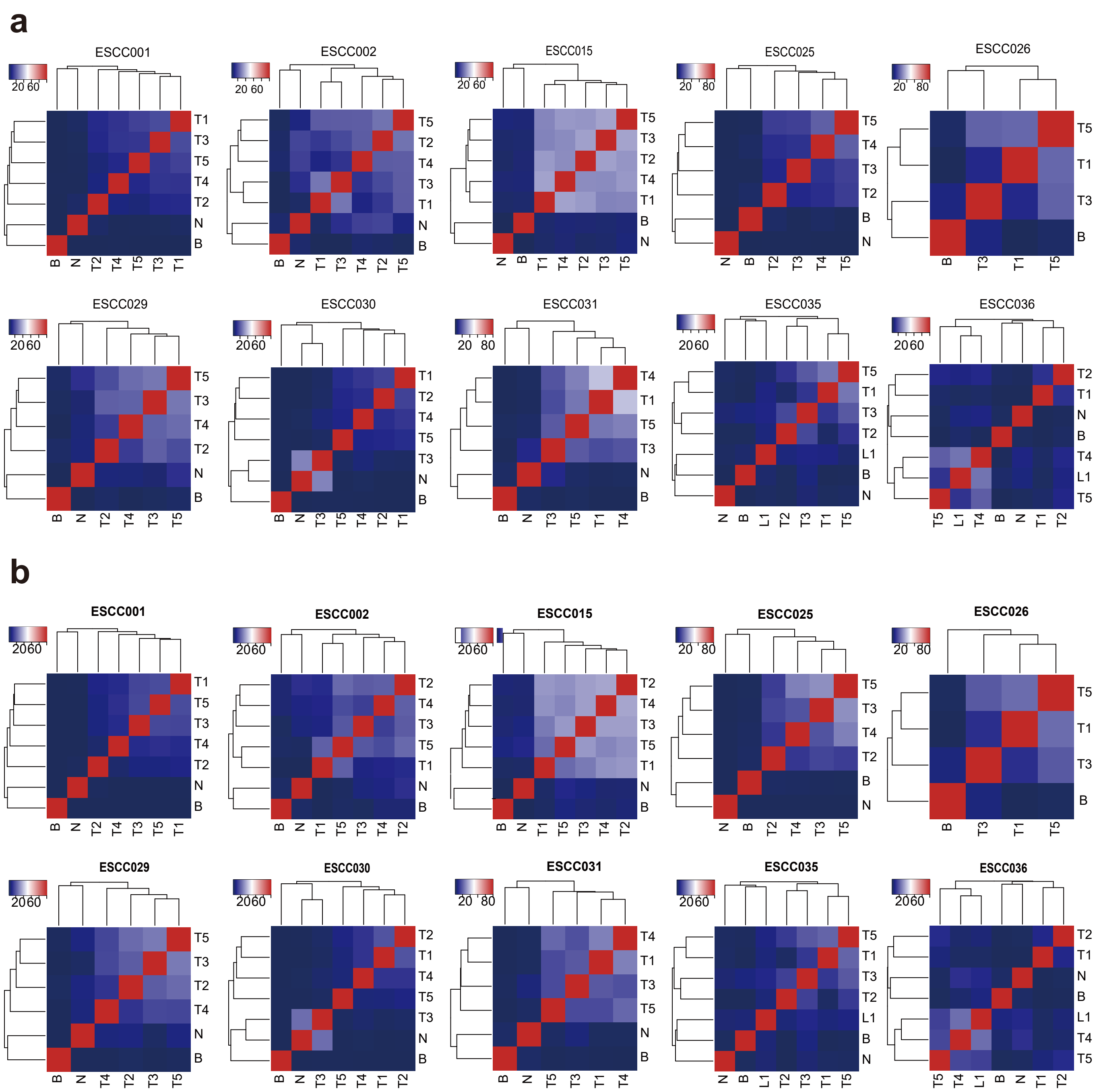

Supplementary Figure 18. The overlapped rate of CDR3 amino acid sequences between any two sample in each patient. (a) TRA. **(b)** TRB. The heatmap shows the results of dendrogram by hierarchical clustering using the correlation coefficients by R (heatmap 3 package).

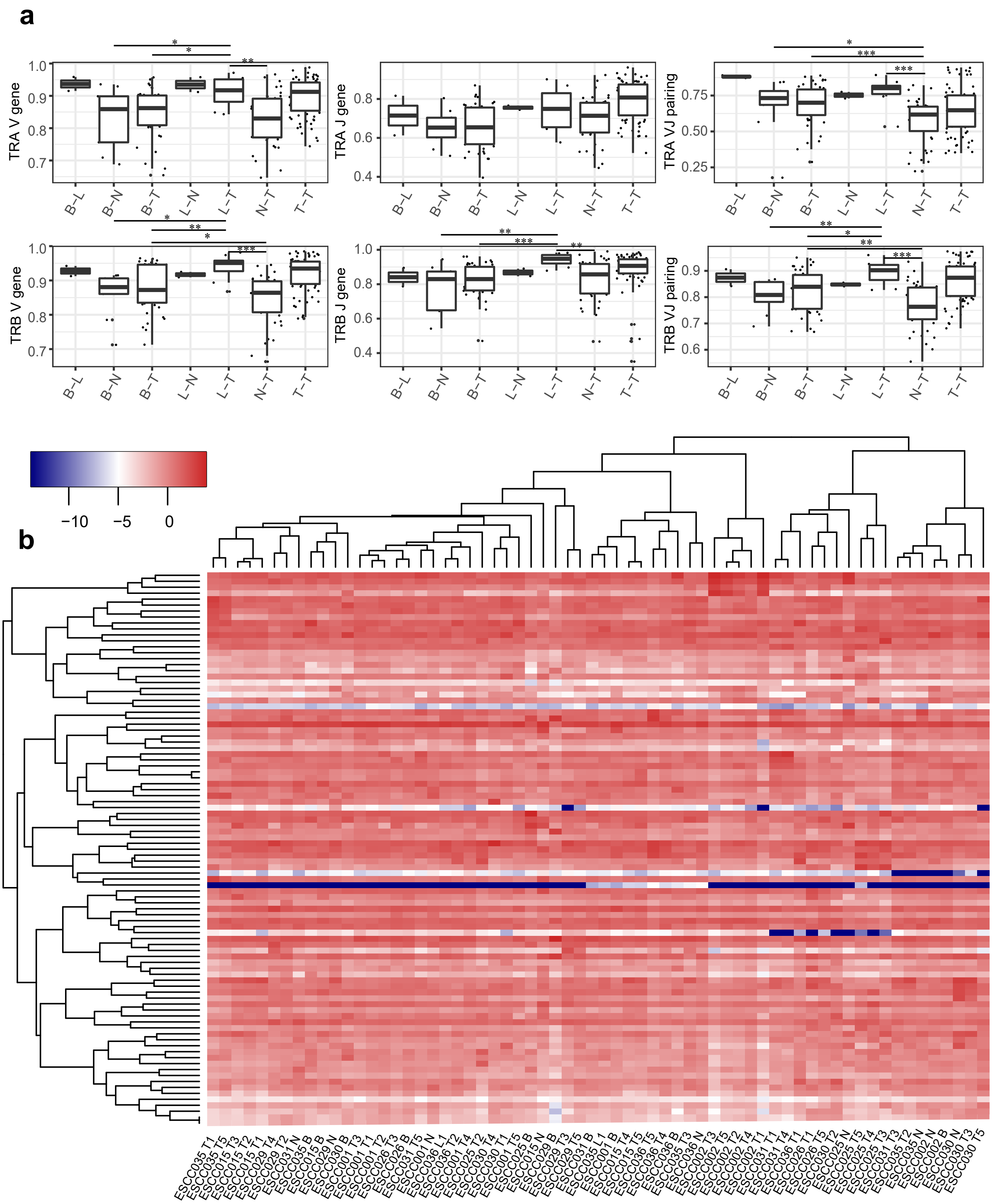

Supplementary Figure 19. Comparison of V gene, J gene and V-J gene pairings. (a) The similarity of genes between two types of samples (top panel: TRA, bottom panel: TRB). The similarity was calculated by Spearman correlation coefficient. The groups B-L, L-N and T-T are excluded for test. **(b)** Heatmap of phenograph clusters of all samples by V gene usage. The y axis present V gene frequencies. The dendrograms for samples and V genes are determined by hierarchical clustering (using R).

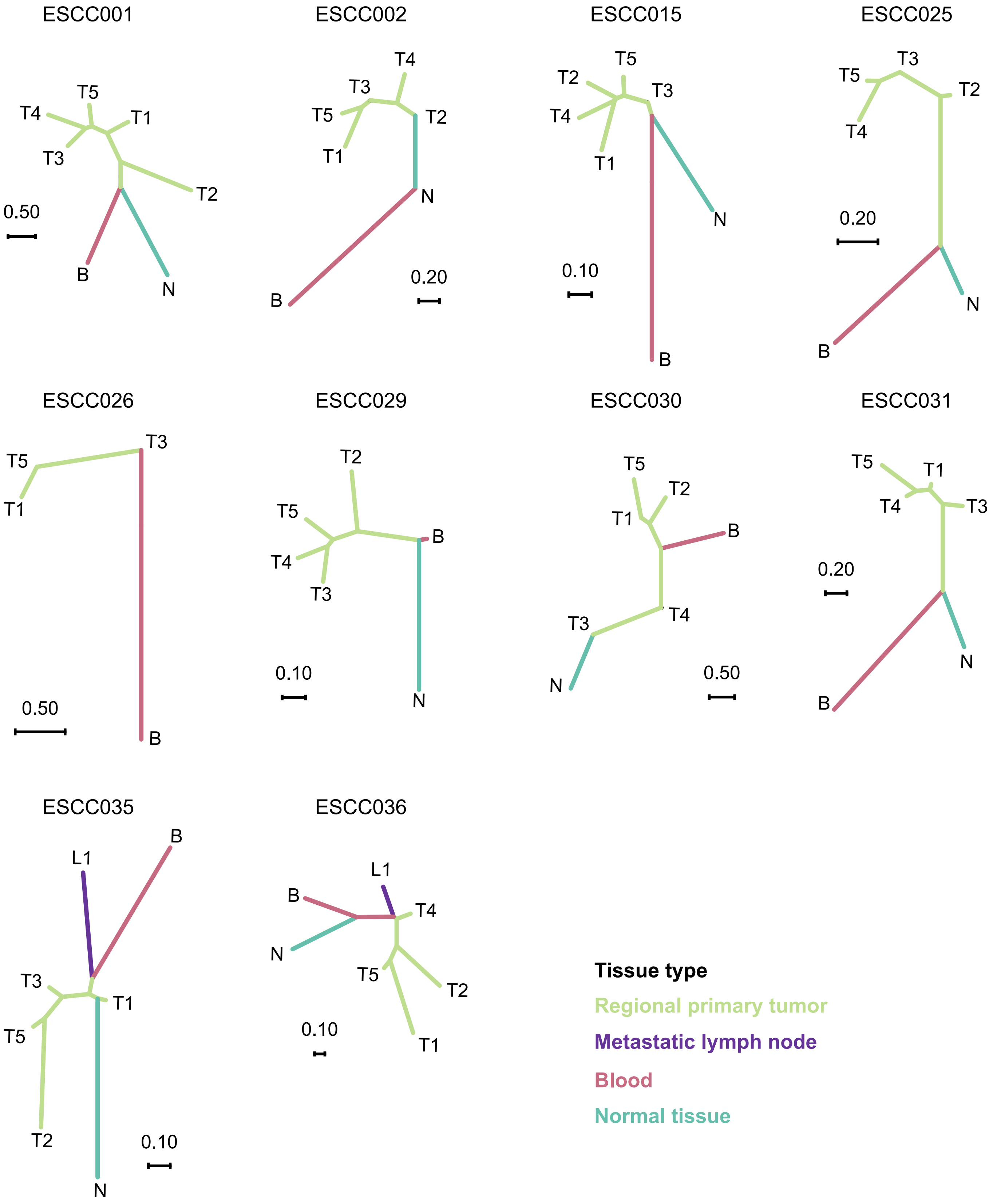

Supplementary Figure 20. Neighbor-joining trees based on TCR data for 10 patients. Pairwise distance among all samples from each patient using TCR repertories overlap to generate a distance metric. A neighbor-joining tree for each patient was constructed using these distances. Trees were color-coded by tissue types.
