# Supplementary material for: Multi-region sequencing unveils novel actionable targets and spatial heterogeneity in esophageal squamous cell carcinoma
Source: Nat Commun. 2019 Apr 11;10:1670. doi: 10.1038/s41467-019-09255-1 (PMC6459928; doi:10.1038/s41467-019-09255-1)
Supplement: Supplementary file 2 — Description of Additional Supplementary Files [file 41467_2019_9255_MOESM2_ESM.pdf]

Description of Additional Supplementary Information

File Name: Supplementary Data 1
Description: Clinical features of 39 ESCC patients.

File Name: Supplementary Data 2
Description: (a) List of somatic coding SNVs and indels of 39 ESCC individuals. (b) List of somatic SNVs of 3 samples in discovery (224 WES) and validation (3 WES) cohort.

File Name: Supplementary Data 3
Description: Focal amplification or deletion of 185 samples from 39 patients. Red color displays different type of amp/del, and blue color represents amp/del rescued by phylogenetic tree.

File Name: Supplementary Data 4
Description: Relevant contributions of 30 COSMIC mutational signatures of 39 ESCC individuals.

File Name: Supplementary Data 5
Description: Mutational signatures identified in 39 ESCCs and split according to timing of mutations (Trunk /Heterogeneous sheet).

File Name: Supplementary Data 6
Description: Statistics of neutral model in 39 ESCCs.

File Name: Supplementary Data 7
Description: Difference of allelic imbalance between spatial samples from the same patient. P-value was given for the statistical significance of the difference.
